# Methyl Syringate: A Primary Driving Factor in Manuka Honeys Ability to Ameliorate Neutrophil Intracellular ROS Activity and NETosis

**DOI:** 10.31083/j.fbl2907255

**Published:** 2024-07-19

**Authors:** Evan N. Main, James C. Huang, Gary L. Bowlin

**Affiliations:** 1 Department of Biomedical Engineering, University of Memphis, Memphis, TN 38152, USA

**Keywords:** neutrophils, NETosis, ROS, host-biomaterial response, inflammation, Manuka honey, biomaterial additives, antioxidants

## Abstract

**Background:**

Neutrophils use both the production of reactive oxygen species (ROS) and a specialized process called NETosis to defend the body from material deemed foreign. While these neutrophil behaviors are critical in preventing infection, a dysregulated response can lead to tissue damage and fibrosis at host-biomaterial interfaces. It was hypothesized that applying the flavonoids found in Manuka honey: chrysin, pinocembrin, and pinobanksin, and the phenolic compound methyl syringate to neutrophils exhibiting pro-inflammatory behavior will reduce ROS activity and prevent NETosis in primary human neutrophils.

**Methods:**

Using primary human neutrophils isolated from donor (n = 5) peripheral blood, concentrations between 1 nM and 10 μM of each flavonoid, 10 μM and 2 mM of methyl syringate, 0.1% v/v and 10% v/v Manuka honey, and combinations of both 1 nM–10 μM of each flavonoid and 10 μM–2 mM of methyl syringate were assayed for reductions in NETosis using Sytox orange extracellular DNA staining and reduction in intracellular ROS activity via standard dichloro-dihydro-fluorescein diacetate (DCFH-DA) oxidation assay.

**Results:**

Compared to positive control levels, individual flavonoids showed moderate effect sizes. Higher concentrations of flavonoids, especially in combination, stimulated ROS activity by up to 105%. Whole Manuka honey reduced neutrophil extracellular trap (NET) levels by up to 91% but only reduced ROS activity by 36%. However, methyl syringate reduced NET levels by up to 68% and ROS activity by 66%.

**Conclusions:**

Methyl syringate and whole Manuka honey are potent inhibitors of neutrophil intracellular ROS activity and NET formation. Methyl syringate potentially drives the anti-inflammatory capabilities of Manuka honey demonstrated by previous studies.

## 1. Introduction

### 1.1 Neutrophil Response to Implanted Biomaterial

The purpose of biomaterials for tissue engineering and regenerative medicine is to regenerate, support, or replace a damaged tissue. To achieve this goal, the biomaterial must first withstand the immune response initiated by the recognition of a foreign material implanted within the body. It is a commonly used saying within the field of biomaterials that “there is no such thing as an inert biomaterial”. However cleverly designed, the device is still deemed an intruder by the innate immune system. This initial immune response, both to the material itself and the injury necessary for implantation, has the potential to be deleterious to proper healing if dysregulated. Commonly referred to as the immune system’s “first responders”, neutrophils quickly swarm to the implant site and interact with soluble blood serum proteins absorbed onto the biomaterial’s surface [1]. These cells, at one time dismissed as short-lived phagocytes with little other function of note, then begin a complex and interconnected process to neutralize potential pathogens, coordinate subsequent steps in the immune response, and pave the way for regeneration of tissue at the implant-host interface [2]. While studies have shown that a neutrophil response is necessary to achieve effective wound healing, an overabundant response can lead to the initiation of chronic inflammation [3]. The development of chronic inflammation leads to delayed and incomplete tissue regeneration and, subsequently, to fibrosis at the tissue-device interface [4].

The host response to a biomaterial implant is a complex and interplaying network of numerous cell types. This response can be broadly summarized in the following phases: initial injury, blood–biomaterial interaction, provisional matrix formation, acute and chronic inflammation, granulation tissue formation, foreign body response, and fibrous encapsulation [5]. Successful resolution of each phase will then positively affect the subsequent phases of the continuum. However, unsuccessful resolution of each phase will shift the continuum away from functional tissue regeneration and toward premature wound closure and the replacement of functional tissue with scar tissue. Thus, a prolonged and overabundant response from neutrophils can be a critical determinant of the end-stage fate of a biomaterial implant.

**Fig. 1.**
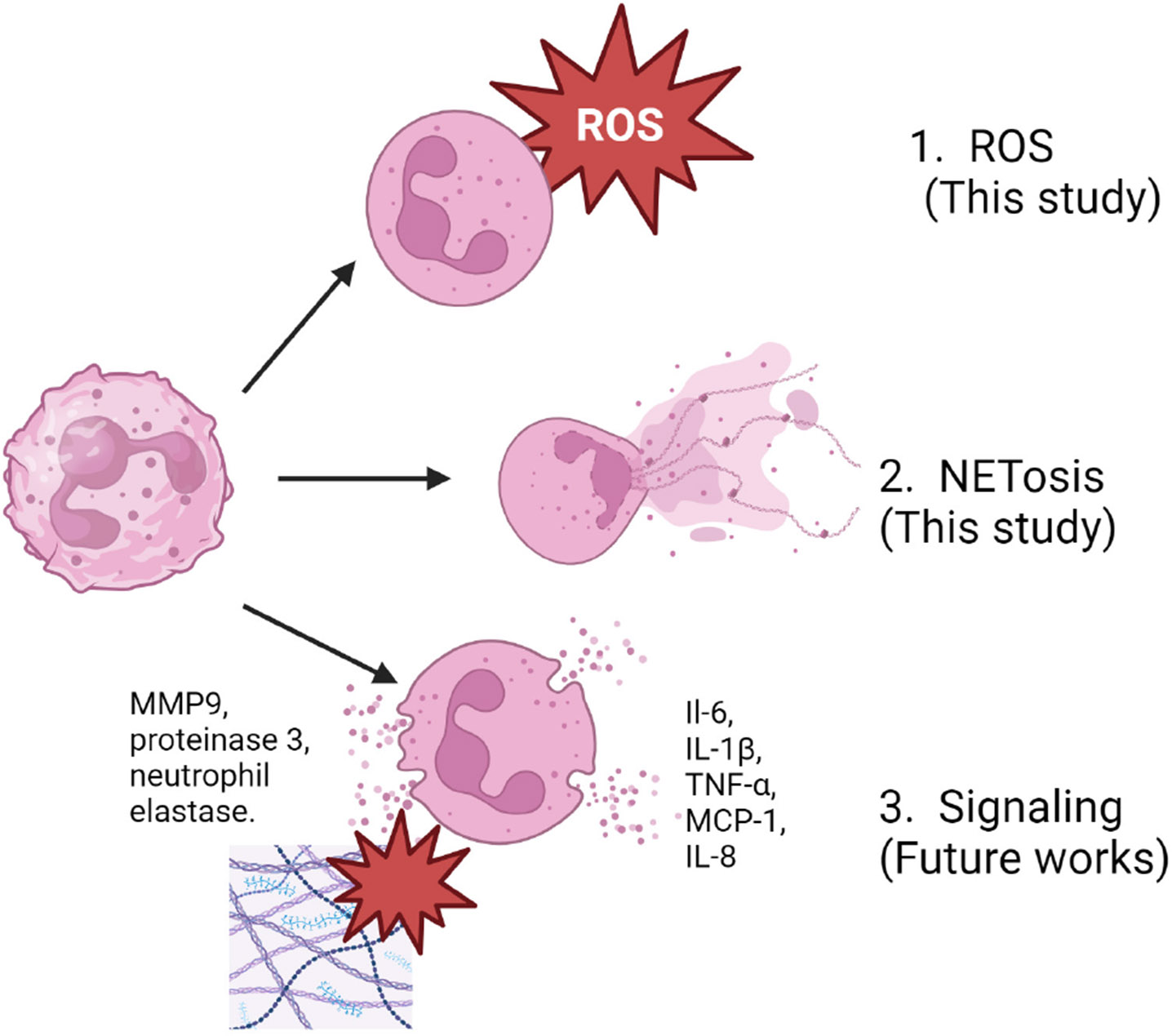
The three broad categories of neutrophil inflammatory behavior. ROS, reactive oxygen species; NETosis, neutrophils can undergo a specialized form of cell death; MMP9, matrix metalloproteinase-9; IL, interleukin; TNF, tumor Necrosis Factor; MCP-1, monocyte chemoattractant protein 1. Created with BioRender.com.

The response of neutrophils to an implanted biomaterial can be broadly categorized in three main ways (Fig. 1). Neutrophils can act as potent signalers, releasing a multitude of pro-inflammatory cytokines and chemokines in response to foreign material. These signals recruit other immune cells and additional neutrophils to swarm to the site of implantation and cause a shift towards pro-inflammatory phenotypes in macrophages and neutrophils arriving in the area. Additionally, neutrophils can undergo a specialized form of cell death called NETosis, wherein they release neutrophil extracellular traps (NETs). These NETs consist of histones entangled within fibrillary, interlinked strands of chromatin, which are coated with proteins derived from the granules of the neutrophil and act to sequester and degrade foreign materials. During an overabundant neutrophil response to an implanted biomaterial, NETs can wall off the biomaterial’s surface and block proper tissue integration. During NETosis, pro-inflammatory signals and degradative enzymes are also released. Neutrophils are also potent generators of Reactive Oxygen Species (ROS), using oxidative stress to damage and degrade foreign materials [2,6]. The signaling, NET forming, and ROS-generating capacities of neutrophils make them highly effective at defending the body from invading pathogens. In the case of biomaterial implantation, however, where the foreign body is not a threat to be eliminated, excessive neutrophil activity can have drastic consequences to the implant microenvironment, driving the continuation of acute inflammation to chronic inflammation. The present study chose to investigate neutrophils’ ROS and NET forming functions, with further studies focusing on the broad array of neutrophil signaling functions.

Neutrophil inflammatory behaviors are highly linked with intracellular ROS production. Heightened ROS levels within the cytoplasm of neutrophils activate inhibitory kinase kinase (IKK), activating the pro-inflammatory NF-*κ*B pathway, increasing the transcription of pro-inflammatory cytokines, recruiting more pro-inflammatory-primed neutrophils and other immune cells to the implant area [7]. Antioxidant therapeutics have been demonstrated to reduce the activation of NF-*κ*B and thus reduce the expression of key inflammatory signals in neutrophils [8]. Similarly, intracellular ROS production is crucial in releasing neutrophil elastase (NE) and myeloperoxidase (MPO) from neutrophil granules during NETosis [9].

### 1.2 Manuka Honey

#### 1.2.1 What It is, What Separates It from Other Honey

Over the past three decades, there has been a resurgence in the investigation into honey as a wound-healing therapeutic. Specifically, Manuka honey has shown great promise due to the presence of methylglyoxal, a potent antibacterial agent that has even shown promise against antibiotic-resistant bacteria. Manuka honey also creates an osmotic potential due to its high sugar content, debriding the wound environment. These unique factors combined help prevent infection and aid healing in the wound microenvironment [10]. In addition to antibacterial and wound cleansing properties, Manuka honey has also been investigated for its anti-inflammatory properties. Previous findings have indicated that Manuka honey lowers neutrophil inflammatory behavior within a therapeutic window [11]. Of particular interest in the field of tissue engineering and regenerative medicine is the fact that these properties can be maintained when Manuka honey is incorporated into fibrous electrospun biomaterials [12].

#### 1.2.2 Previous Findings in the Role of Manuka Honey in Wound Healing

Previous findings have shown that Manuka honey reduces activation of the inflammatory NF-*κ*B signal pathway, chemotaxis, and release of several key inflammatory cytokines in a dHL-60 cell line neutrophil model [13,14]. Pilot study data have shown that Manuka honey reduces NETosis and MMP-9 output within a therapeutic window in primary human neutrophils *in vitro*. While the mechanisms by which whole Manuka honey inhibits neutrophil inflammatory behavior are still not fully understood, Manuka honey contains antioxidant flavonoid components such as pinobanksin, pinocembrin, chrysin, and methyl syringate. These components have demonstrated the capacity to scavenge ROS and reduce MPO activity [15,16]. Intracellular ROS production and MPO activation are important upstream inflammation signals and activators of NETosis. To bring Manuka honey closer towards clinical viability as a biomaterial additive, it must first be elucidated which components of Manuka honey are responsible for its antiinflammatory and antioxidant effects.

**Fig. 2.**
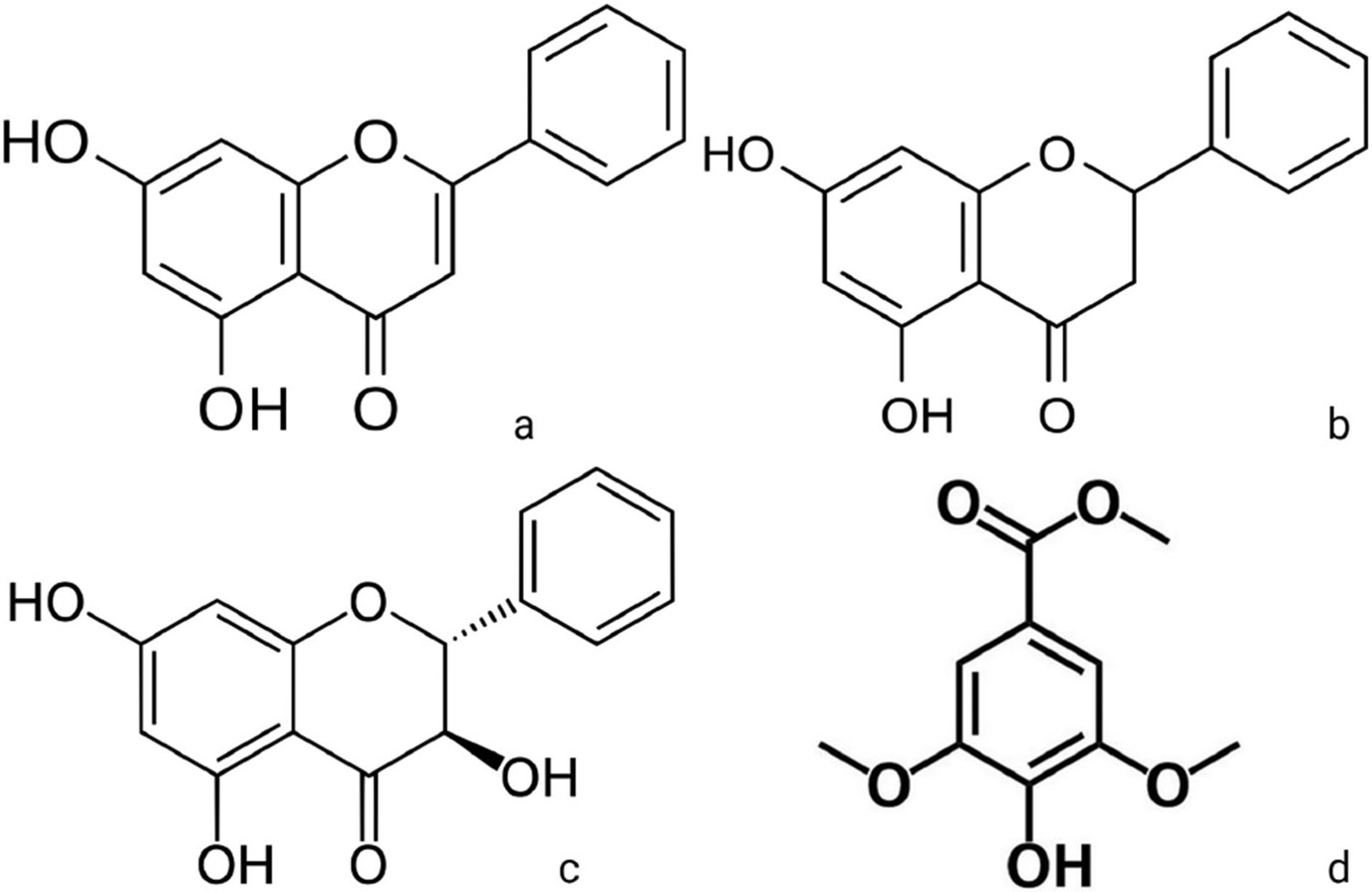
Major flavonoids found in Manuka honey. (a) Chrysin, (b) pinocembrin , and (c) pinobanksin and (d) phenolic component methyl syringate. Created with BioRender.com.

Antioxidants found in Manuka honey, such as pinobanksin, pinocembrin, and chrysin, could reduce the secretion of pro-inflammatory signals resulting from NF-*κ*B activation and NETosis [17,18]. The cytoskeletal reorganization during NETosis is highly dependent on MPO initiating NE release. Methyl syringate has shown promise in inhibiting MPO, potentially preventing NETosis from stimulated neutrophils [19]. Pinobanksin, pinocembrin, chrysin, and methyl syringate (Fig. 2) are found in Manuka honey and comprise a majority of Manuka honey’s flavonoid and phenolic content. Previous findings indicate that Manuka honey reduces extracellular ROS production, chemotaxis, activation of the NF-*κ*B pathway, and release of pro-inflammatory cytokines in a differentiated neutrophil-like HL-60 model [13]. These antioxidant and MPO-inactivating components are hypothesized to be responsible for the anti-inflammatory effects that Manuka honey demonstrated on neutrophils, but these mechanisms have yet to be confirmed.

The compounds selected to investigate as antiinflammatory therapeutics are pinobanksin, pinocembrin, chrysin, and methyl syringate. This selection is due to the fact that they are the most abundant flavonoids in Manuka honey [20,21]. Methyl syringate was included as it was found to comprise approximately 70% w/w of the phenolic fraction of Manuka honey [22]. The cytotoxicity limit of honey was determined to be at concentrations above 5% v/v [14].

The effects of whole Manuka honey have shown promise on neutrophil inflammatory behavior in dHL60 models and some primary human neutrophil studies [11,13,14,23]. The present study aimed to expand on these results by elucidating the components within Manuka honey responsible for these effects and further delving into the composition of components that yield the best results regarding ROS generation and NET formation.

Another goal of the present study was to determine if the flavonoid/phenolic content of Manuka honey can be more effective than Manuka honey as a whole and to circumvent the cytotoxic limit. Animal studies have demonstrated these components to be non-toxic up to a concentration of at least 15 mg per kg body weight [24-29]. Utilizing these bioactive agents found in prominence in Manuka honey to reduce ROS generation and NETosis, the present study aimed to elucidate primary agents involved in Manuka honey’s ability to reduce neutrophil inflammation. The next phase of this study sought to determine whether these agents act best outside of whole Manuka honey or if a complementary/synergistic effect is seen from the compounds within whole Manuka honey.

In the present work, activated primary human neutrophils isolated from donor peripheral blood were utilized to screen serial concentrations of pinobanksin, pinocembrin, chrysin, and methyl syringate to determine which flavonoids/phenolic components and at what concentration ranges are most effective in reducing intracellular ROS levels and NET release. Additionally, serial concentrations of whole Manuka honey for reductions in intracellular ROS levels and NET release were screened. It was then determined the therapeutic ranges for each Manuka honey component. Additional investigation was performed on whether a combination “cocktail” of multiple active components or whole Manuka honey would be more efficacious for incorporating into biomaterials to prevent excessive neutrophil inflammation upon implantation.

Pinobanksin, pinocembrin, and chrysin concentrations in Manuka honey are on the order of 100–500 nM, and previous findings from this lab have determined the therapeutic window of Manuka honey to be between 0.1–1% v/v, corresponding to 0.1–5 nM of the flavonoids [14,20]. Serial concentrations between 1 nM and 10 μM of each flavonoid were tested to determine if these compounds are effective even at ranges outside what is found in honey. The concentration of Methyl syringate within Manuka honey is widely variable even within the same study, ranging between 27 nmol per gram of Manuka honey and 1048 nmol per gram of Manuka honey [30]. Thus, a wide range of 10 μM to 2 mM was adopted for this study.

## 2. Materials and Methods

### 2.1 Neutrophil Isolation Procedure and Culture

For each selected Manuka honey component, five separate experiments were conducted using fresh peripheral blood neutrophils isolated from 5 individual donors of randomized sex (with precautions taken to ensure all donors are not the same sex) using a well-established method in accordance with protocols approved by the University of Memphis Institutional Review Board (IRB ID: #PRO-FY2020-230) including informed consent [31-34]. Primary neutrophil inflammatory responses can vary based on sex, age, weight, and underlying disease [35-38]. Additionally, Nonsteroidal anti-inflammatory drugs (NSAIDs), blood sugar fluctuations, and alcohol consumption can affect neutrophil activity [39-41]. Studying these effects is not a focus of this work at this phase, so randomized numbers of male and female donors (avoiding homogenous data sets) were utilized, and donors were restricted to non-smoking, under 30 years of age with no history of endocrine, autoimmune, cardiovascular disease, or inflammatory diseases. Donors were instructed to avoid NSAIDs and alcohol consumption 48 hours before donation and to avoid caffeine and eating within 12 hours of blood donation.

Blood was collected from donors using K2 EDTA-coated vacutainers (BD, Franklin Lakes, NJ, USA, ref. no. # 366643) to prevent coagulation and chelate calcium (a critical signaling ion for many neutrophil inflammatory pathways) to prevent premature activation of neutrophils [42]. Autologous serum was collected in a separate untreated serum tube (BD, ref. no. # 366668) to maintain sufficient divalent ion concentrations in the serum. Neutrophils were isolated using a long-standing procedure validated in previous works from this lab and from publications from other labs. The purity of neutrophils from this method of isolation is reported at above 96% [32,43-45]. Briefly, blood was sedimented into a white blood cell segment and a red blood cell segment. The white blood cell segment was aspirated and centrifuged at 200 ×g for 10 minutes at room temperature using a Sorvall ST8 Centrifuge (Rotor ID: 75005701, Thermo Scientific, Waltham, MA, USA). The supernatant was aspirated, the pellet was resuspended in PBS (pH 7.4, Gibco, Waltham, MA USA, ref. no. 10010-023) overlaid onto 3 mL Isolymph (CTL, Deer Park, NY, USA, density 1.077 ± 0.001 g/mL, ref. no. 759050) and centrifuged at 300 ×g at room temperature using the Sorvall ST8 Centrifuge (Rotor ID: 75005701, Thermo Scientific) for 40 minutes at room temperature with soft acceleration and deceleration. The monocyte layer was carefully removed to avoid contamination, and the supernatant was aspirated. The pellet was resuspended in 4 °C hypotonic (0.2%) NaCl solution for 30 seconds to lyse the remaining red blood cells. Tonicity was restored with 1.6% NaCl solution at 4 °C. NaCl solutions were made using ACS grade Sodium Chloride (MP Biomedicals, Santa Ana, CA, USA, ref. no.194738) dissolved into sterile, endotoxin-free cell culture grade water (Cytiva, Marlborough, MA, USA, ref. no. SH30529.02). Cells and red blood cell lysate were centrifuged at 200 ×g for 7 minutes at 4 °C temperature using a Sorvall ST8 Centrifuge (Rotor ID: 75005701, Thermo Scientific) and then resuspended and washed in 4 °C PBS. Isolated neutrophils were then resuspended in 4 °C Hank’s buffered salt solution without calcium and magnesium (HBSS, Gibco, ref. no. 14175-095) containing 0.2% autologous human serum and 10 mM N-2-hydroxyethylpiperazine-N-2-ethane sulfonic acid (HEPES, Corning, Corning, NY, USA, ref. no. 25-060-CI) and counted using a Countess II FL automated cell counter (Thermo Fisher). Cell viability and purity were also confirmed post-isolation using the Countess II FL automated cell counter and 0.4% Trypan blue (Gibco, ref. no. 15250-061) exclusion.

After isolation, neutrophils were seeded at 100,000 cells per well in a 96-well black plate with clear bottom (MidSci, Fenton, MO, USA, ref. no. 781671) in 150 μL HBSS without calcium and magnesium, 10 mM HEPES, and 0.2% autologous serum. The cells were then stimulated with 100 nM phorbol-12-myristate-13-acetate (PMA, Sigma Aldrich, Saint Louis, MO, USA, ref. no. P8139). PMA is an artificial stimulus which activates protein kinase C (PKC) pathways, MPO, and neutrophil elastase (NE). Isoform PKC *β*, activated by PMA, triggers PI3K*γ* pathway, which also activates ERK, contributing to neutrophil ROS production. Thus, PMA is known to induce neutrophil degranulation, ROS production, and NETosis in nanomolar concentrations [46]. Chrysin (Thermo Scientific, ref. no. 110320050), pinocembrin (Sigma Aldrich product no. P5239), pinobanksin (Sigma Aldrich ref. no. 68530), and methyl syringate (Sigma Aldrich, ref. no. S409448) were obtained in pure (*>*99%) powder form and stock solutions dissolved in dimethyl sulfoxide (DMSO, Fisher Chemical, ref. no. BP231-100). Each Manuka honey component was added according to the dilution ranges mentioned previously (1 nM and 10 μM for flavonoids and 10 μM to 2 mM for methyl syringate). Additionally, serial dilutions of whole Manuka honey between 0.1% and 10% (Manuka-Gard, Monterey, CA, USA, MGO 400, ref. no. MG2232-203) were assayed. All sample groups and control groups were seeded in quadruplicate.

After cell seeding, addition of Manuka honey components or whole Manuka honey, and pro-inflammatory stimulus with PMA, neutrophils were incubated until terminal timepoints at 3 and 6 hours with positive control (PMA-stimulated) and negative controls (non-stimulated cells with 0.1% DMSO to correspond with the highest concentration of DMSO present in flavonoid dilutions in addition to PMA solutions). Neutrophils were incubated at 37 °C, 5% CO_2_, and 100% humidity in a sterile incubator.

### 2.2 NETosis

**Fig. 3.**
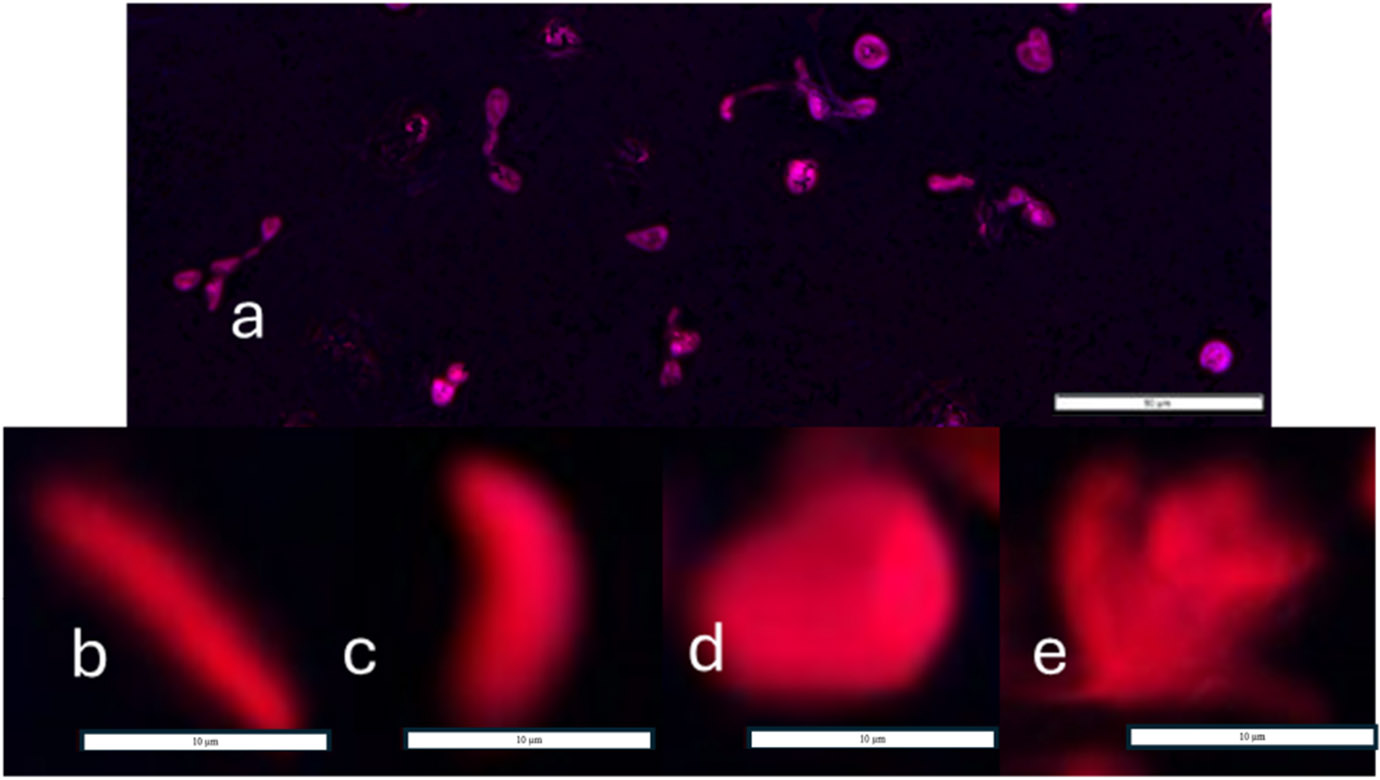
Sytox orange stains neutrophil extracellular trap (NET)-associated extracellular DNA in stimulated neutrophils. (a) Blue fluorescence from DAPI staining of nuclei at 40 × magnification. Scale Bar = 50 μM. (b–e) Varying phases of NET release from stimulated neutrophils. Scale Bar = 10 μM.

At 3- and 6-hour time points, using a validated lab protocol, isolated neutrophils were stained with 0.25 μM Sytox Orange (Invitrogen, Waltham, MA, USA ref. no. S11368) and incubated at room temperature in the dark for 5 minutes. Sytox orange is a cell-impermeant nucleic acid stain. Thus, Sytox orange was utilized to stain NET-associated extracellular DNA. NET associated extracellular DNA is characterized by it’s decondensation and diffusion away from the cell as stained by Sytox orange (Fig. 3), whereas necrosis is characterized with intact condensed nuclei and apoptotic cells maintain cellular integrity (not allowing diffusion of Sytox stain into the cell to stain nucleic acids) [47]. The plates were centrifuged at 200×g for 5 minutes using a Sorvall Legend XTR Centrifuge (Rotor ID: 75003624, Thermo Scientific). After centrifugation, 100 μL supernatant was removed to reduce background fluorescence. The results were quantified using a SpectraMax i3 spectrophotometer (Molecular Devices, San Jose, CA, USA) at fluorescence excitation and emission wavelengths of 540 and 580, respectively [48]. Wells with 0.25 μM Sytox Orange in 150 μL HBSS, 10 mM HEPES, and 0.2% autologous serum without cells were used as background blank wells to be subtracted from the sample data. All data were normalized to the positive controls per each donor (due to high donor-to-donor variability in neutrophil inflammatory responses) and expressed as mean and standard deviation percent NETs of each experiment (n = 5).

### 2.3 Intracellular ROS Activity

**Fig. 4.**
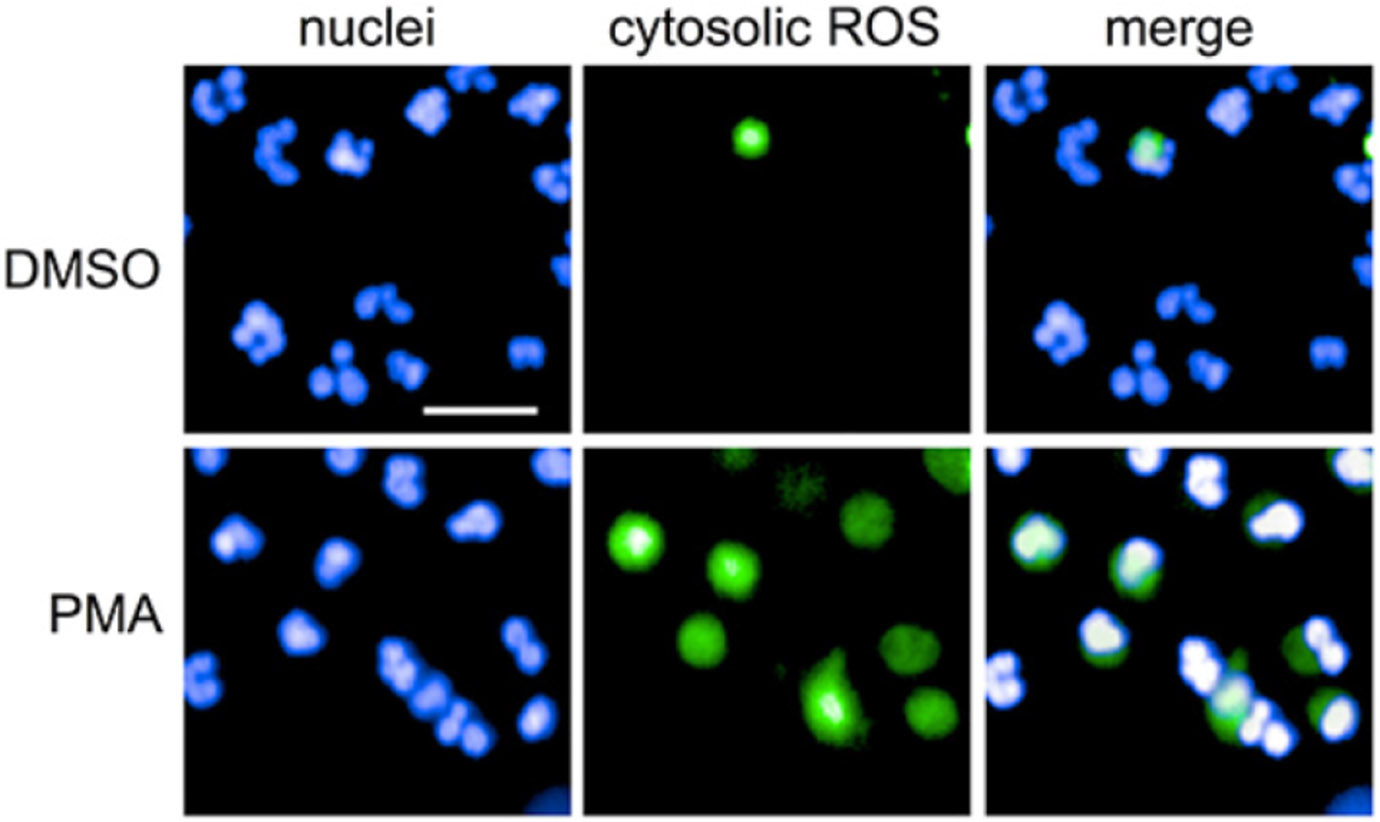
Intracellular ROS as measured by DCFDA. Scale Bar = 30 μM. ROS: Reactive Oxygen Species; DCFDA: 2′,7′–dichlorofluorescein diacetate; DMSO: Dimethylsulfoxide; PMA: phorbol 12-myristate 13-acetate. Reproduced with permission via creative commons license from Sondo E *et al*. Frontiers in Immunology. Published by Frontiers Media SA 2019; 10: 963 [49].

After isolation and prior to cell seeding, stimulus, and exposure to Manuka honey or its components, neutrophils were stained with 2′,7′–dichlorofluorescein diacetate (DCFDA, Abcam Waltham, MA, USA, ref. no. ab113851) which is deacetylated by intracellular esterases to a non-fluorescent compound, and then oxidized by hydroxyl, peroxyl and other ROS into 2′,7′–dichlorofluorescin (DCF), a highly fluorescent compound (Fig. 4, Ref. [49]). Intracellular ROS activity was measured using a microplate assay adapted from the protocol from the manufacturer and a publication from Nadesalingam Ajantha *et al*. [50]. Briefly, isolated neutrophils were suspended at a concentration of 1.5 million cells per mL in HBSS without calcium and magnesium, 10 mM HEPES, and 0.2% autologous serum containing 5 μM DCFDA stain. The neutrophils were then incubated in this solution for 30 minutes in the dark at 37 °C. Following the incubation period, the cells were centrifuged for seven minutes at 200×g using a Sorvall ST8 Centrifuge (Rotor ID: 75005701, Thermo Scientific) at room temperature. The supernatant was removed, and the cells were resuspended in 15 mL of cold PBS (pH 7.4) to wash off residual DCFDA stain. The cell solution was then centrifuged at 200 ×g using the same centrifuge and rotor as the previous centrifugation step. After the supernatant was removed, the cells were resuspended in HBSS without calcium and magnesium, 10 mM HEPES, and 0.2% autologous serum. The cells were then diluted to a concentration of μillion cells per mL and seeded into plates for a final concentration of 100,000 cells per well and then treated with the same protocols listed before in the section titled “Neutrophil isolation procedure and culture” (treated with the selected bioactives, stimulated with PMA, and incubated at 37 °C, 5% CO_2_, and 100% humidity in a sterile incubator). At 3 and 6 hours, plates were read using the spectrophotometer at an excitation and emission wavelength of 485 nm and 535 nm, respectively, in accordance with manufacturer protocol. All data were normalized to the positive controls per each donor (due to high donor-to-donor variability in neutrophil inflammatory responses) and expressed as mean and standard deviation percent intracellular ROS activity per each experiment (n = 5).

### 2.4 Statistics

*A-priori* power analyses were performed to ensure that the performed studies had sufficient sample sizes to provide power values above 80%. As previously stated, all sample groups and controls were performed in quadruplicate per each plate, with five separate experiments using independent blood donors being performed. Using Microsoft excel (Version 2405, Microsoft, Redmond, WA, USA), all data from each experiment was normalized to the positive control for that specific donor. All data were then expressed as percent ROS activity and NET formation compared to donor positive control and then pooled for statistical analysis and data visualization. Since several data sets did not pass the assumptions for normality (according to a Shapiro-Wilk test), non-parametric tests were used.

Due to the extensive data sets generated by this study, graphing and statistical analyses were performed using a custom MATLAB (Version MATLAB 9.7 R2019b, MathWorks, Natick, MA, USA) code. Briefly, using this code, the raw data underwent background subtraction and the positive and negative means were then determined from the data. The individual data concentrations were indexed from the data set into an array of corresponding concentrations. The average value from each concentration was then divided by the average positive control to determine the ratio between them. Using the function on MATLAB, the standard deviation for each concentration was determined. The data were then plotted on a box plot with the four well means of each individual experiment included as data points on a dot plot. Included in each graph for individual Manuka honey components was the most effective Manuka honey concentration data to serve as visual reference and to compare the effect size of the individual concentrations versus whole Manuka honey. Steps one through five were repeated for the 6-hour time point data. Wilcoxon rank sum tests were performed comparing the NET and ROS percent reduction of each concentration group to the positive control group percentage, on an alpha value of 0.05. To verify and quality control the MATLAB code, data condensing and statistics on sample groups were additionally run on Microsoft Excel and the wilcox.test function in R (Version 4.3.0, R Foundation, Indianapolis, IN, USA).

## 3. Results

### 3.1 Flavonoids and Methyl Syringate

#### 3.1.1 NET Formation

**Fig. 5.**
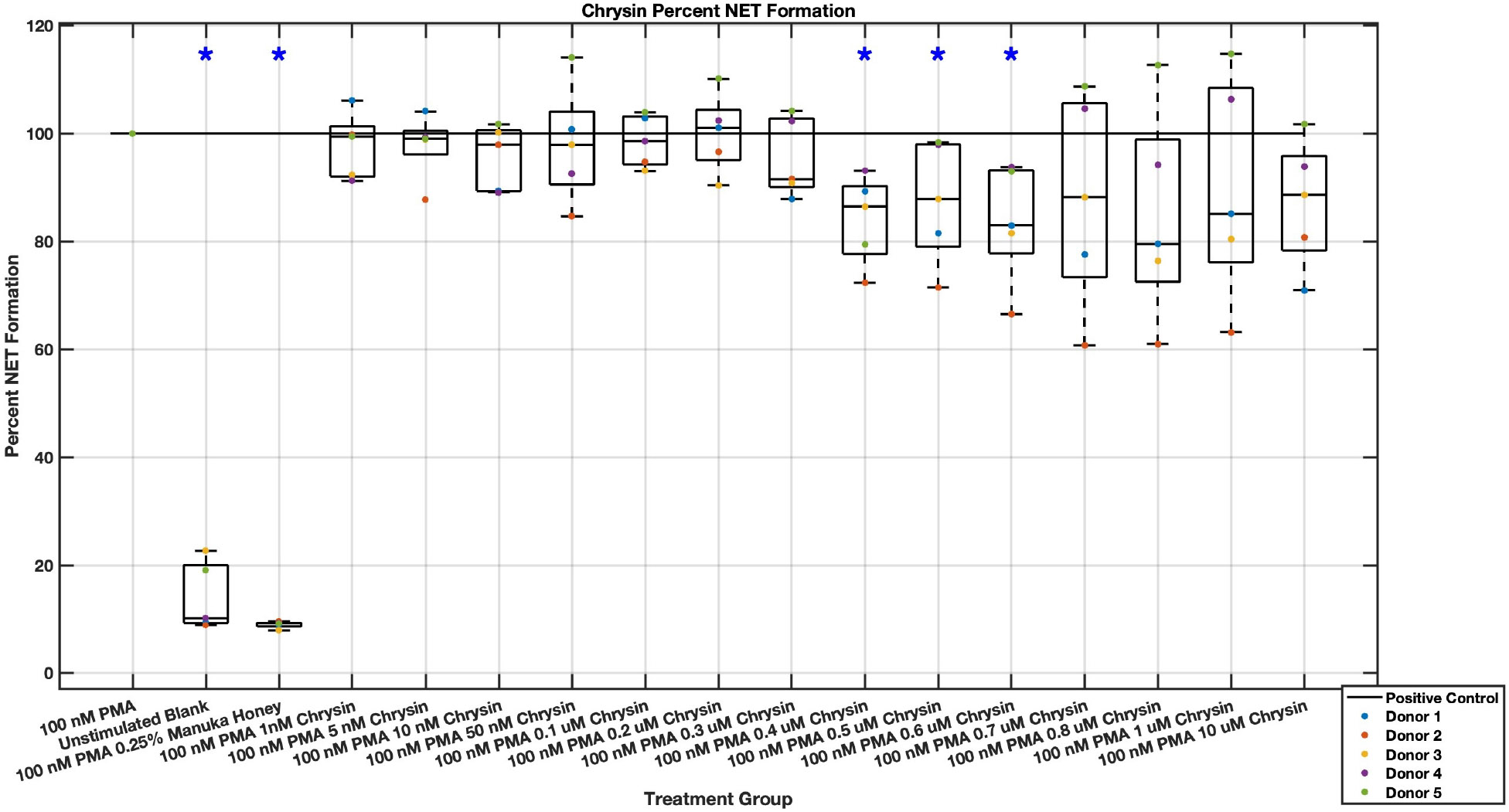
Effect of Chrysin on neutrophil NET formation three hours post-PMA-stimulus. Dot plot points are represented as the mean of four wells for each five experiments (n = 5). Asterisk represents statistical significance from the 100 nM PMA positive control group. *p < 0.05.

**Fig. 6.**
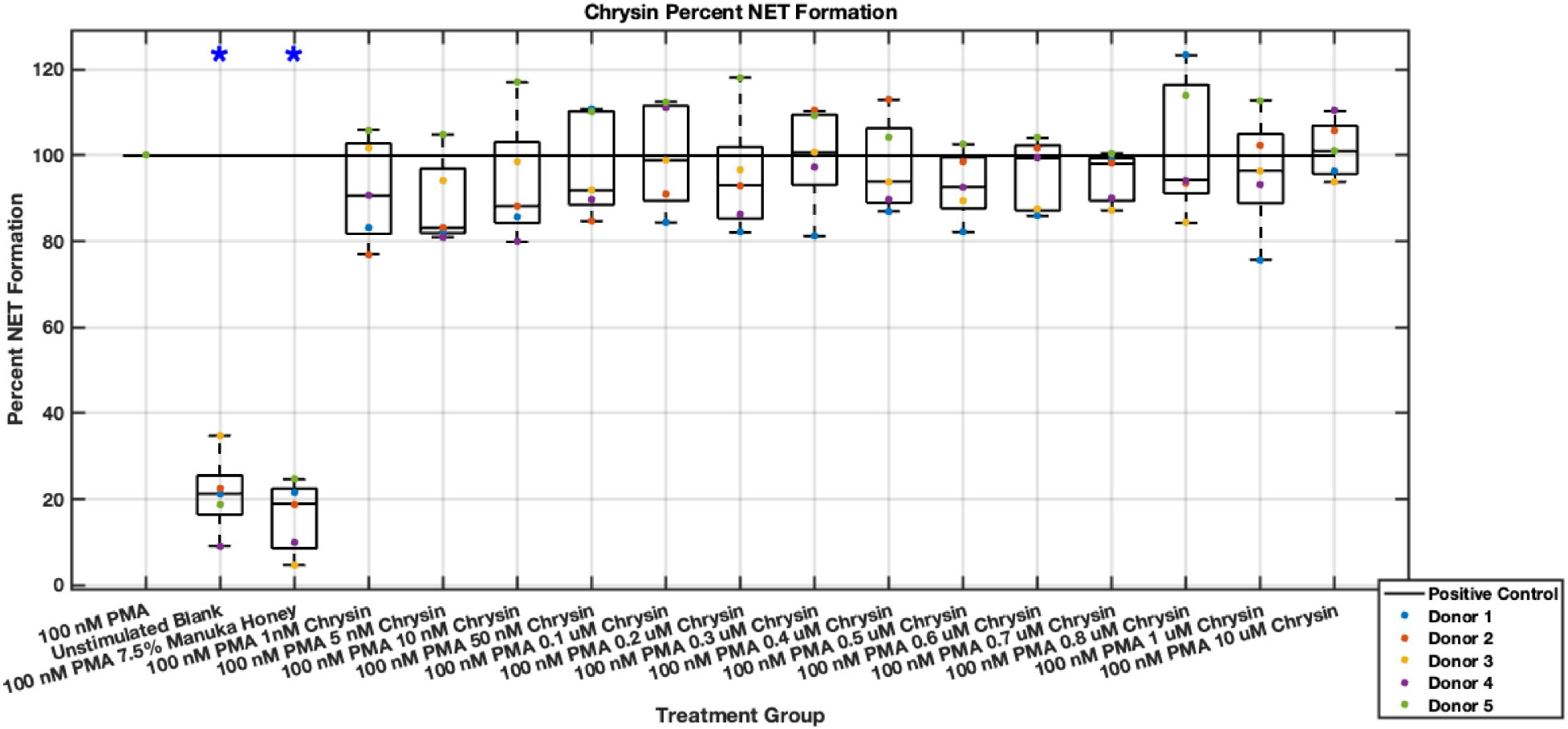
Effect of Chrysin on neutrophil NET formation six hours post-PMA-stimulus. Dot plot points are represented as the mean of four wells for each five experiments (n = 5). Asterisk represents statistical significance from the 100 nM PMA positive control group. *p < 0.05.

**Fig. 7.**
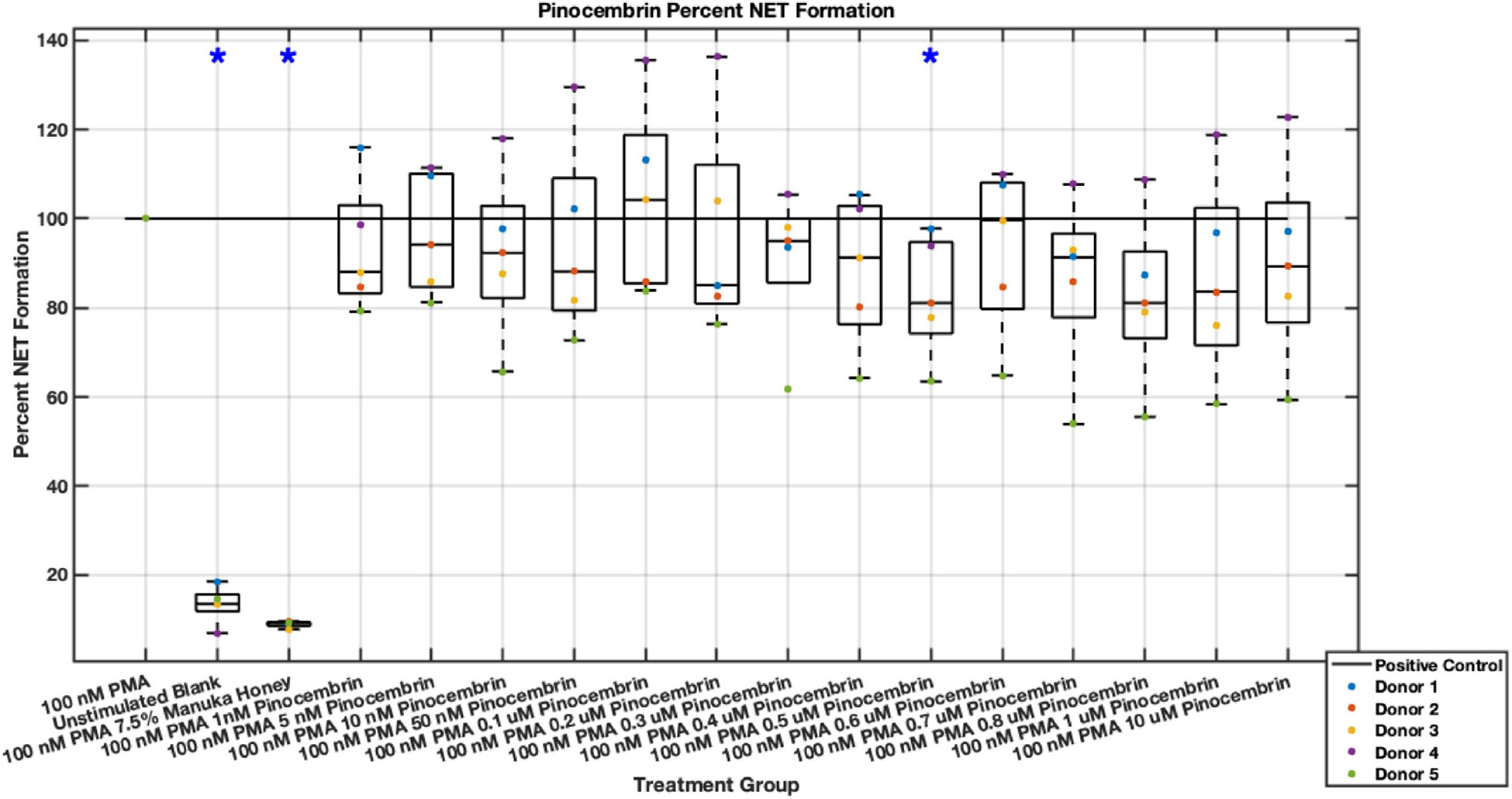
Effect of Pinocembrin on neutrophil NET formation three hours post-PMA-stimulus. Dot plot points are represented as the mean of four wells for each five experiments (n = 5). Asterisk represents statistical significance from the 100 nM PMA positive control group. *p < 0.05.

**Fig. 8.**
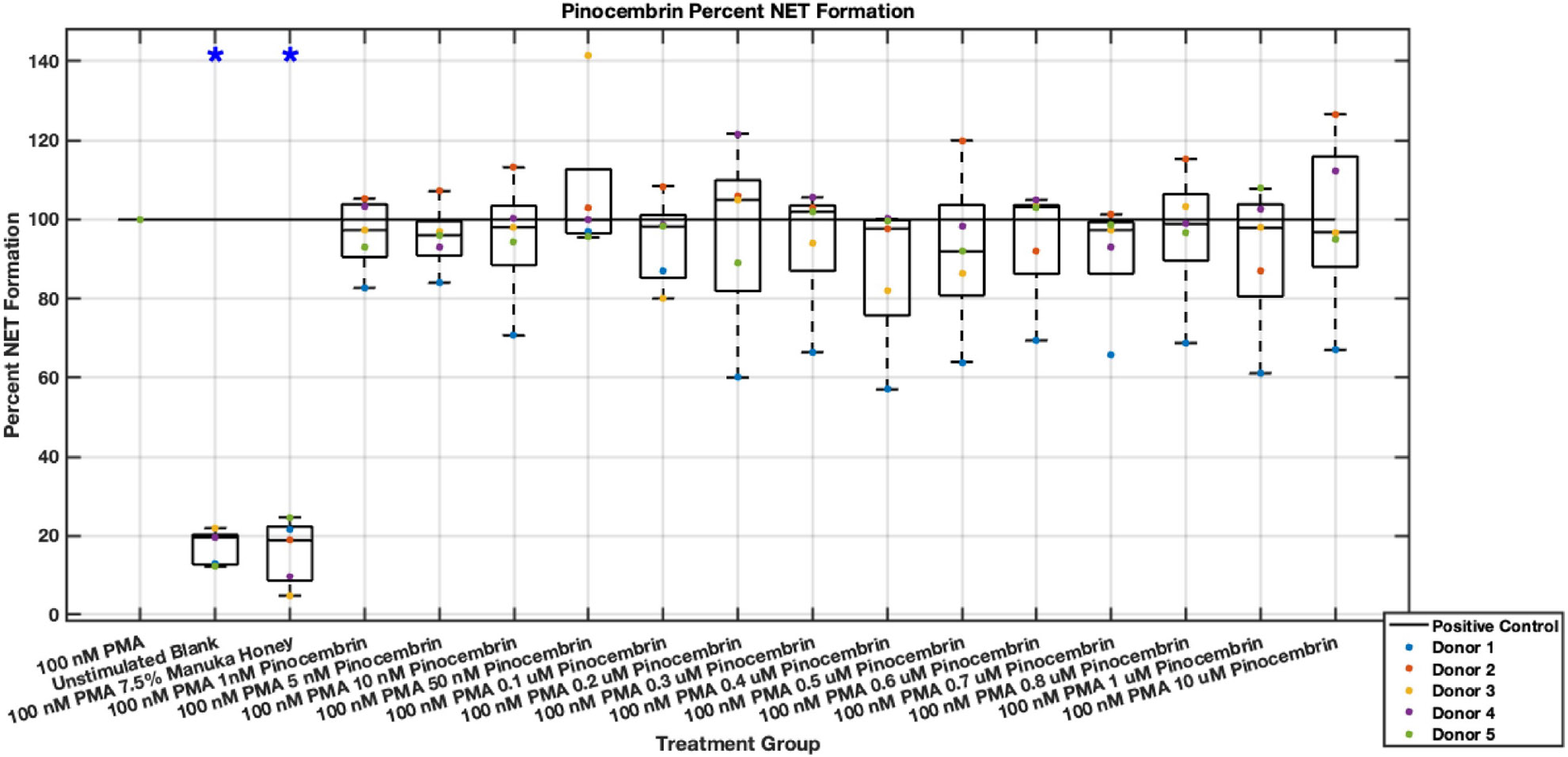
Effect of Pinocembrin on neutrophil NET formation six hours post-PMA-stimulus. Dot plot points are represented as the mean of four wells for each five experiments (n = 5). Asterisk represents statistical significance from the 100 nM PMA positive control group. *p < 0.05.

**Fig. 9.**
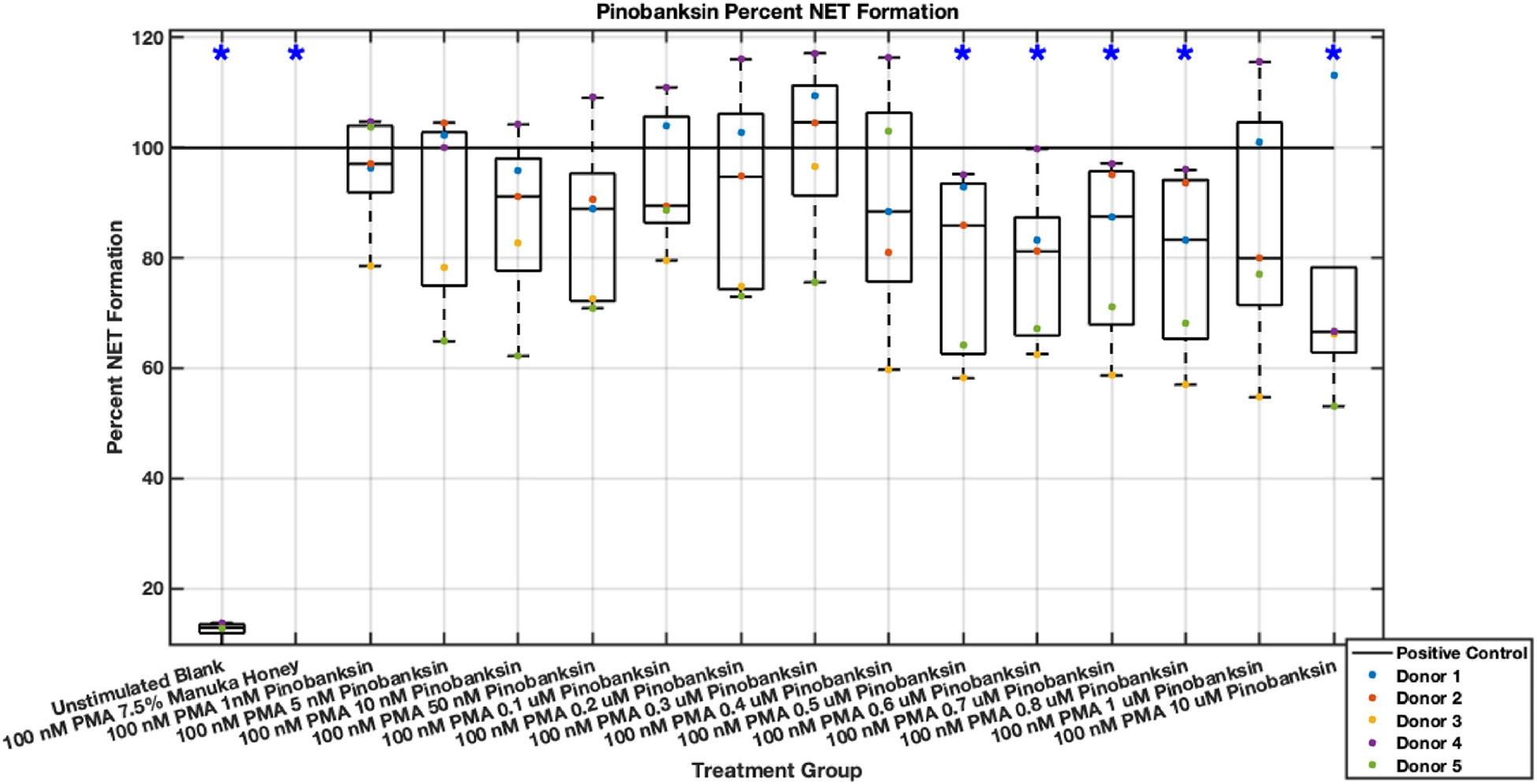
Effect of Pinobanksin on neutrophil NET formation three hours post-PMA-stimulus. Dot plot points are represented as the mean of four wells for each five experiments (n = 5). Asterisk represents statistical significance from the 100 nM PMA positive control group. *p < 0.05.

**Fig. 10.**
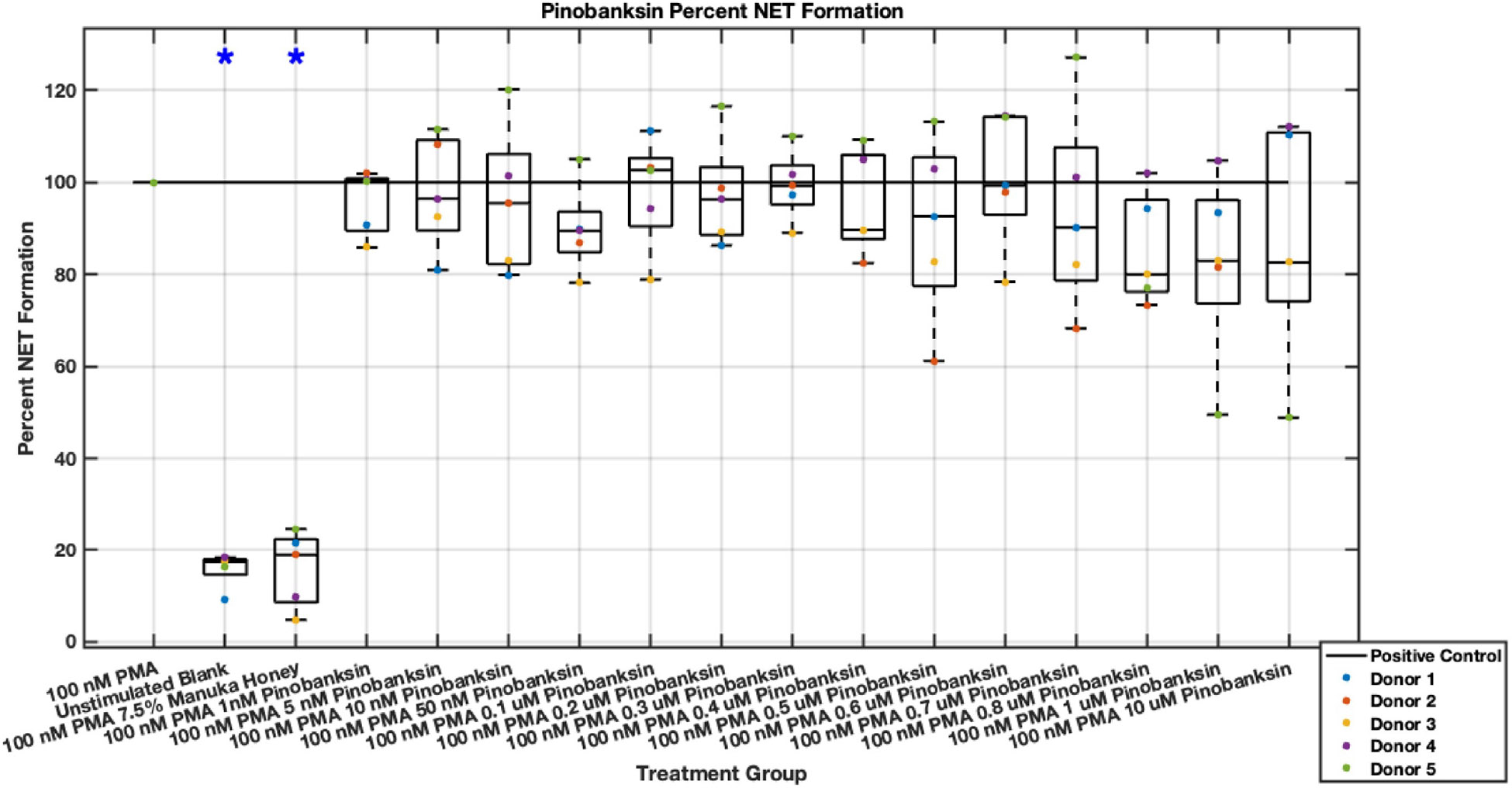
Effect of Pinobanksin on neutrophil NET formation six hours post-PMA-stimulus. Dot plot points are represented as the mean of four wells for each five experiments (n = 5). Asterisk represents statistical significance from the 100 nM PMA positive control group. *p < 0.05.

**Fig. 11.**
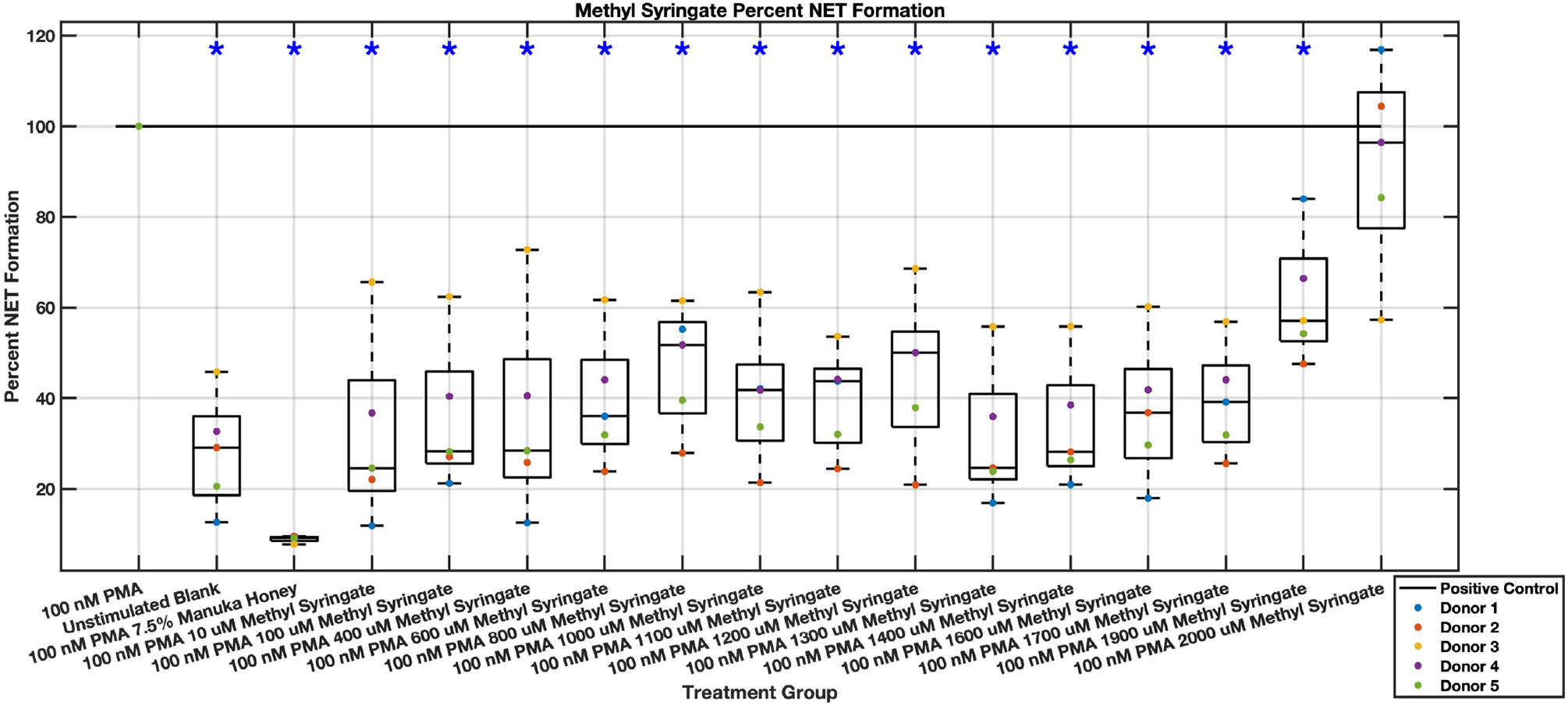
Effect of Methyl Syringate on neutrophil NET formation three hours post-PMA-stimulus. Dot plot points are represented as the mean of four wells for each five experiments (n = 5). Asterisk represents statistical significance from the 100 nM PMA positive control group. *p < 0.05.

**Fig. 12.**
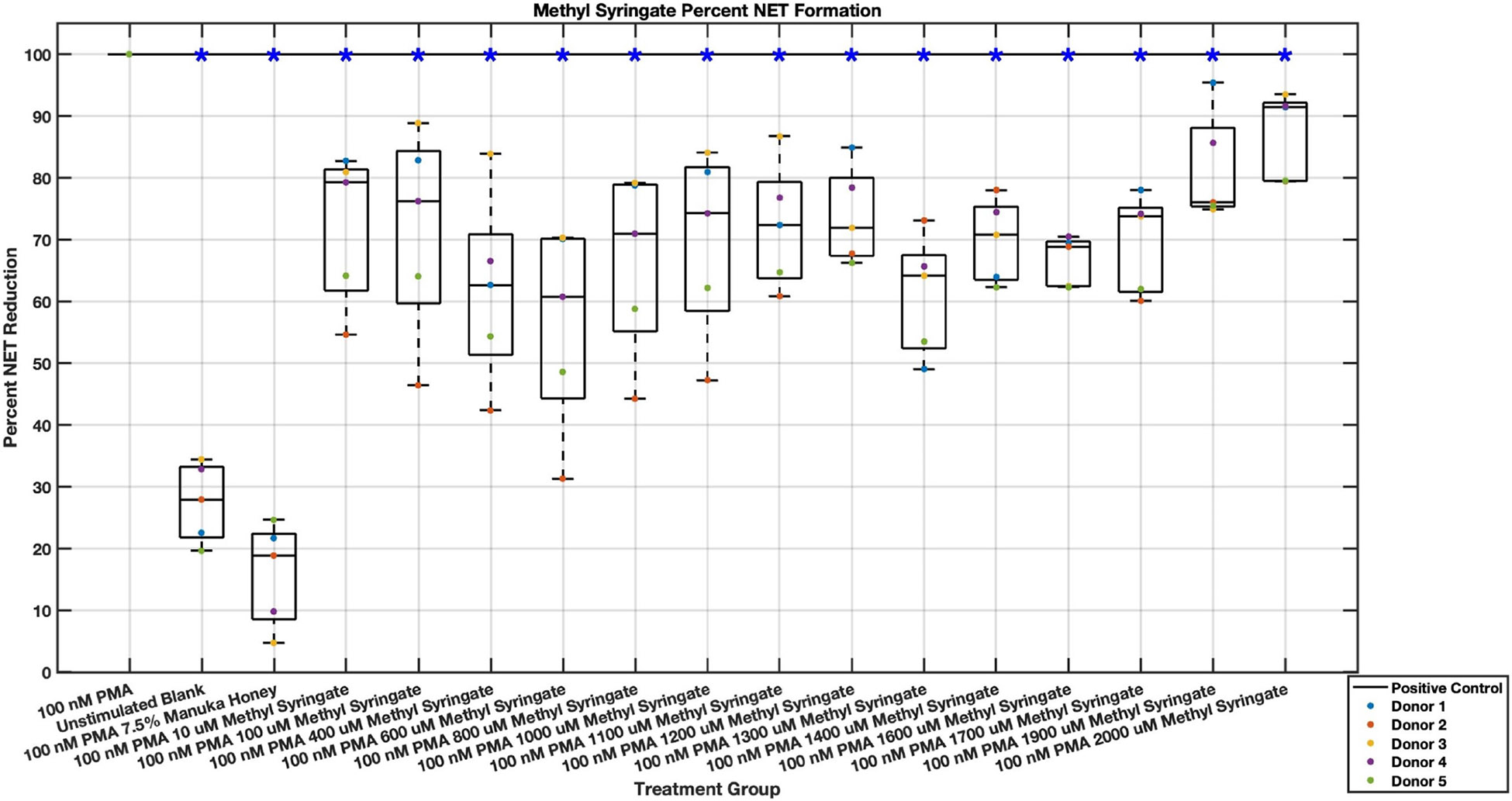
Effect of Methyl Syringate on neutrophil NET formation six hours post-PMA-stimulus. Dot plot points are represented as the mean of four wells for each five experiments (n = 5). Asterisk represents statistical significance from the 100 nM PMA positive control group. *p < 0.05.

Individual flavonoids did not induce a large effect on NET formation in activated neutrophils with median values within 30% of positive control in pinocembrin and pinobanksin data sets (Figs. 5,6,7,8,9,10), but phenolic component methyl syringate shows a robust inhibitory effect on NET release (Figs. 11,12). However, while variance was still present in the data, methyl syringate showed a large effect size, specifically in the 100 μM to 1700 μM range at 3 hours post-stimulation. These data indicate an over 60% decrease in median NET DNA release due to methyl syringate beginning at 100 nM methyl syringate. The highest degree of reduction was observed in 1300 μM methyl syringate, which showed NET levels at 37% of the positive control. Overall, the range of 100 μM to 1700 μM corresponded to median values of 37% to 50% of the positive control, indicating reductions in NETosis of 50% to 63%. A lowering of this effect size and loss of statistical significance in some concentrations was observed after 6 hours post-stimulation, but with similar trends being represented, with a 30% reduction at 1600 μM methyl syringate being statistically significant from the positive control.

#### 3.1.2 ROS Activity

**Fig. 13.**
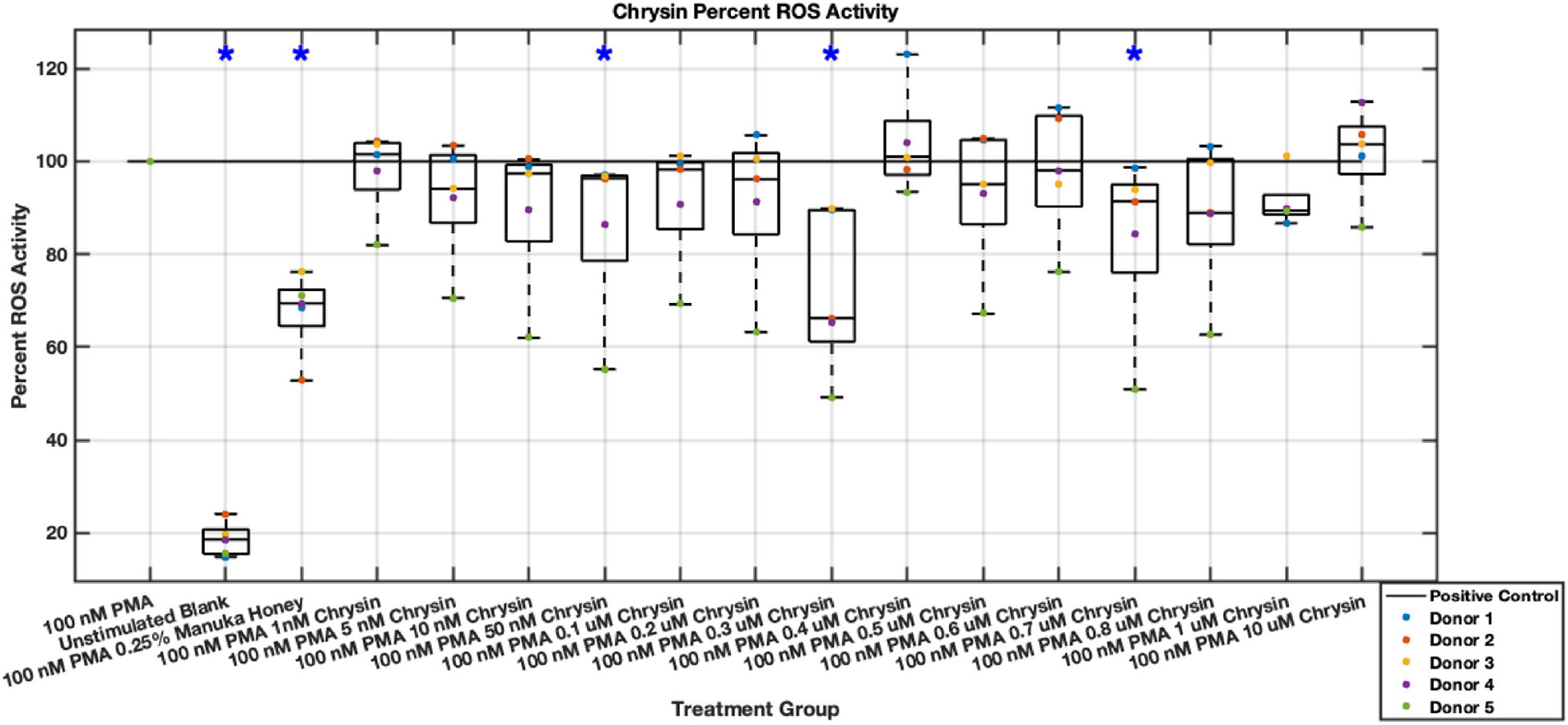
Effect of Chrysin on neutrophil intracellular ROS activity three hours post-PMA-stimulus. Dot plot points are represented as the mean of four wells for each five experiments (n = 5). Asterisk represents statistical significance from the 100 nM PMA positive control group. *p < 0.05.

**Fig. 14.**
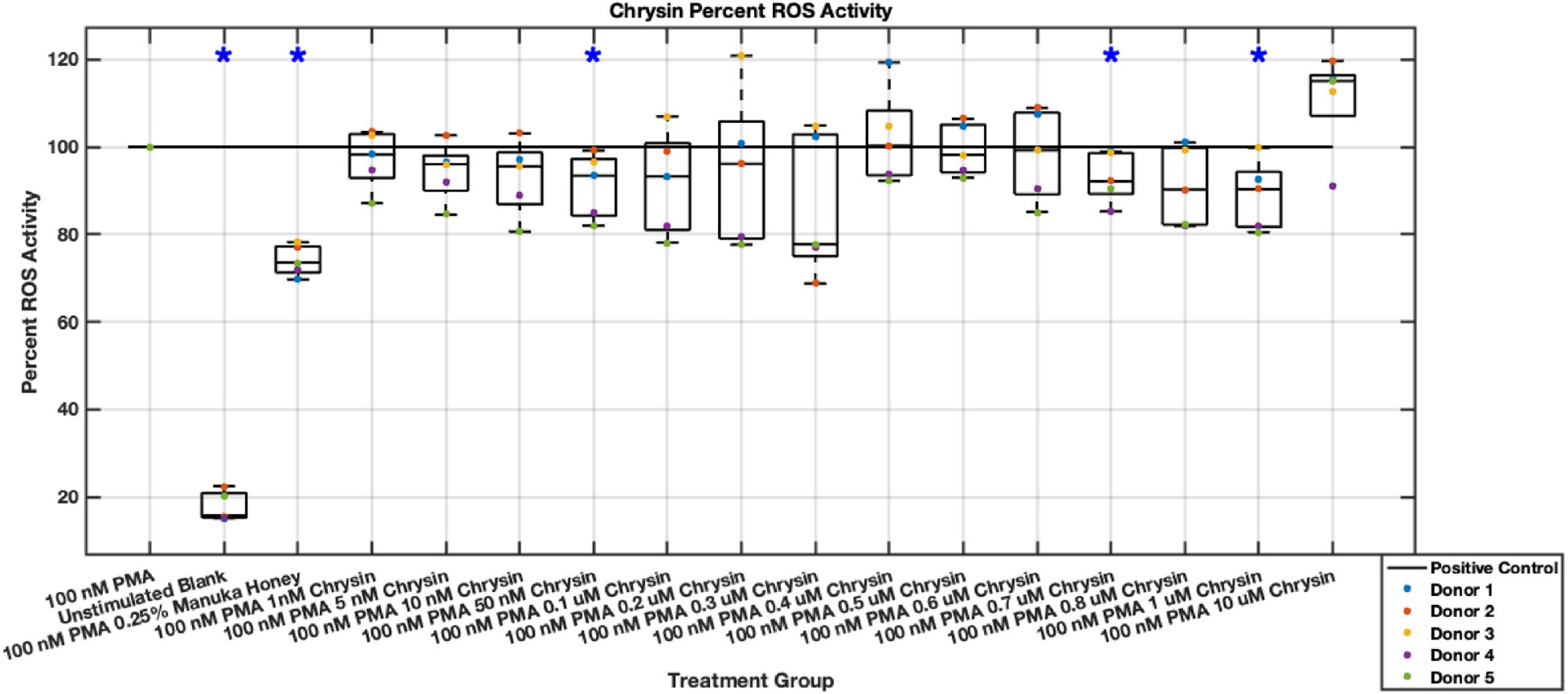
Effect of Chrysin on neutrophil intracellular ROS activity six hours post-PMA-stimulus. Dot plot points are represented as the mean of four wells for each five experiments (n = 5). Asterisk represents statistical significance from the 100 nM PMA positive control group. *p < 0.05.

**Fig. 15.**
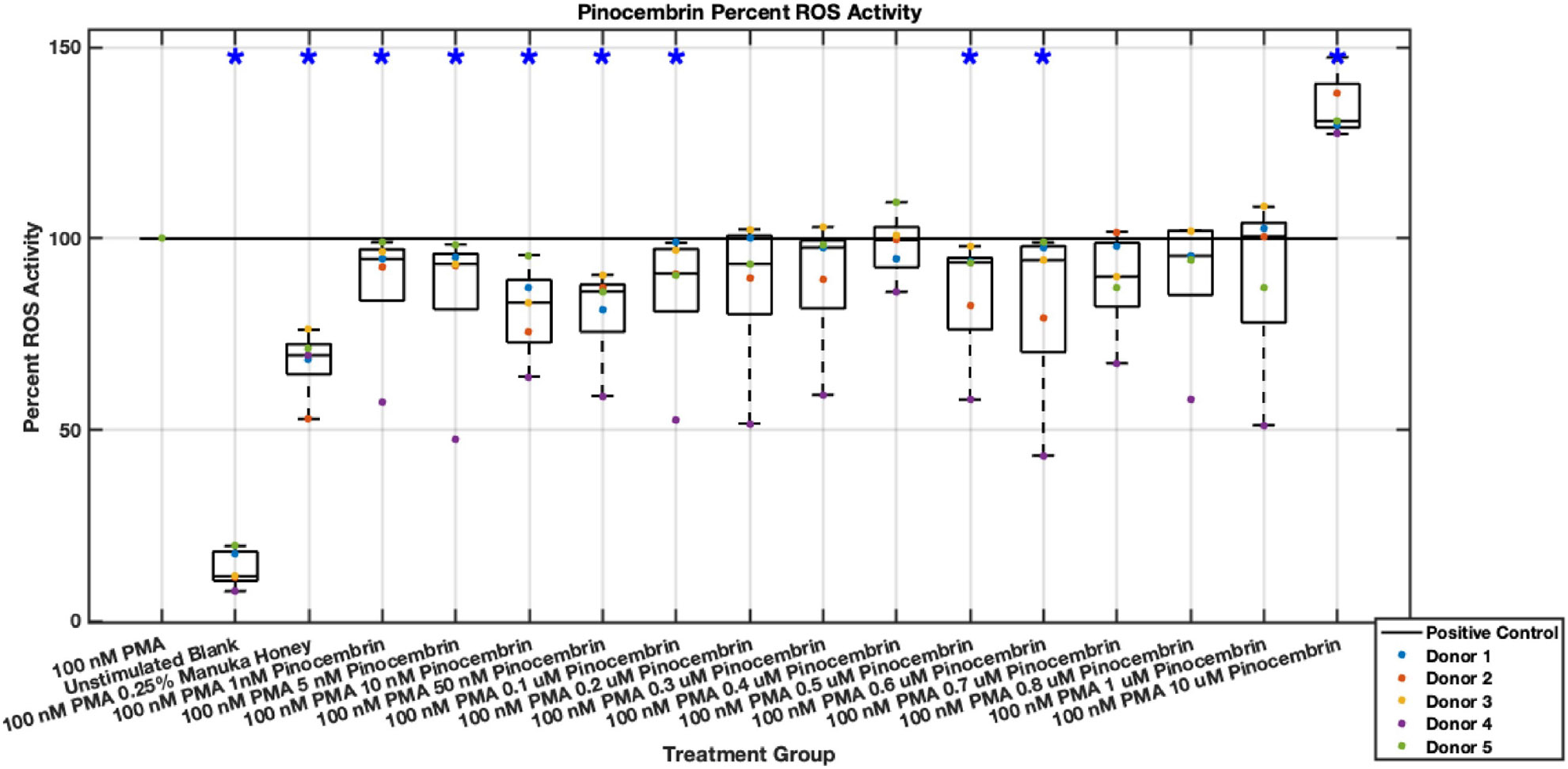
Effect of Pinocembrin on neutrophil intracellular ROS activity three hours post-PMA-stimulus. Dot plot points are represented as the mean of four wells for each five experiments (n = 5). Asterisk represents statistical significance from the 100 nM PMA positive control group. *p < 0.05.

**Fig. 16.**
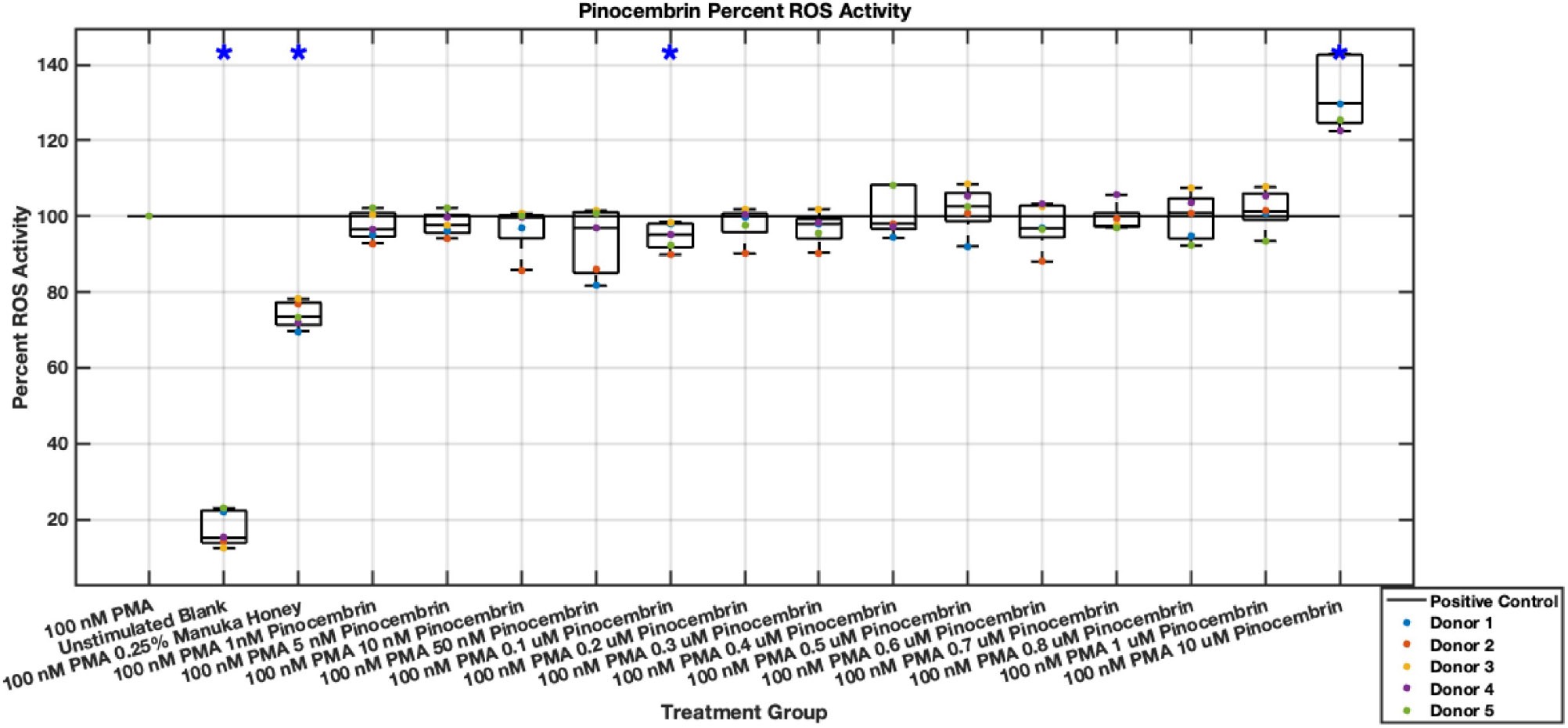
Effect of Pinocembrin on neutrophil intracellular ROS activity six hours post-PMA-stimulus. Dot plot points are represented as the mean of four wells for each five experiments (n = 5). Asterisk represents statistical significance from the 100 nM PMA positive control group. *p < 0.05.

**Fig. 17.**
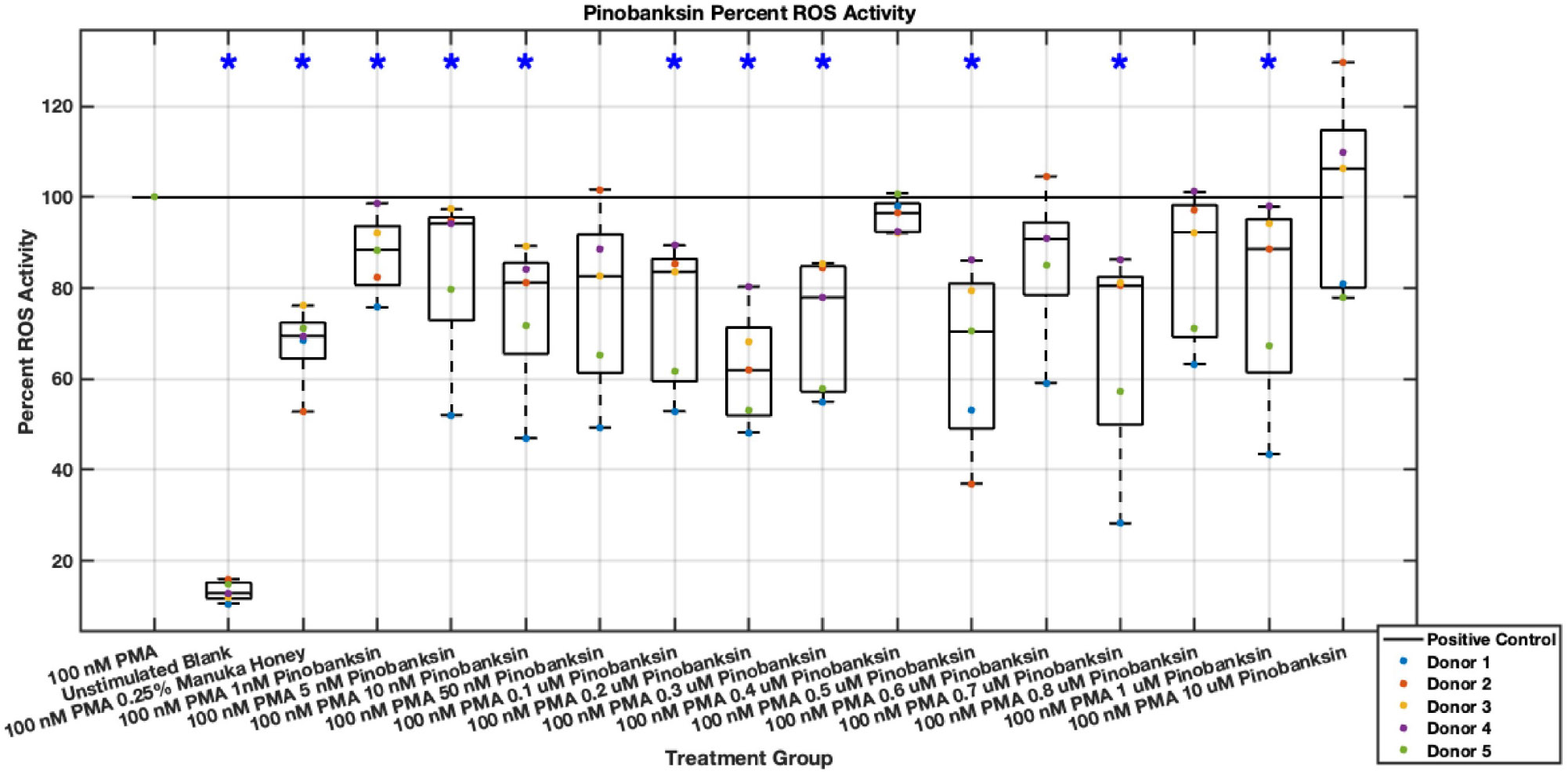
Effect of Pinobanksin on neutrophil intracellular ROS activity three hours post-PMA-stimulus. Dot plot points are represented as the mean of four wells for each five experiments (n = 5). Asterisk represents statistical significance from the 100 nM PMA positive control group. *p < 0.05.

**Fig. 18.**
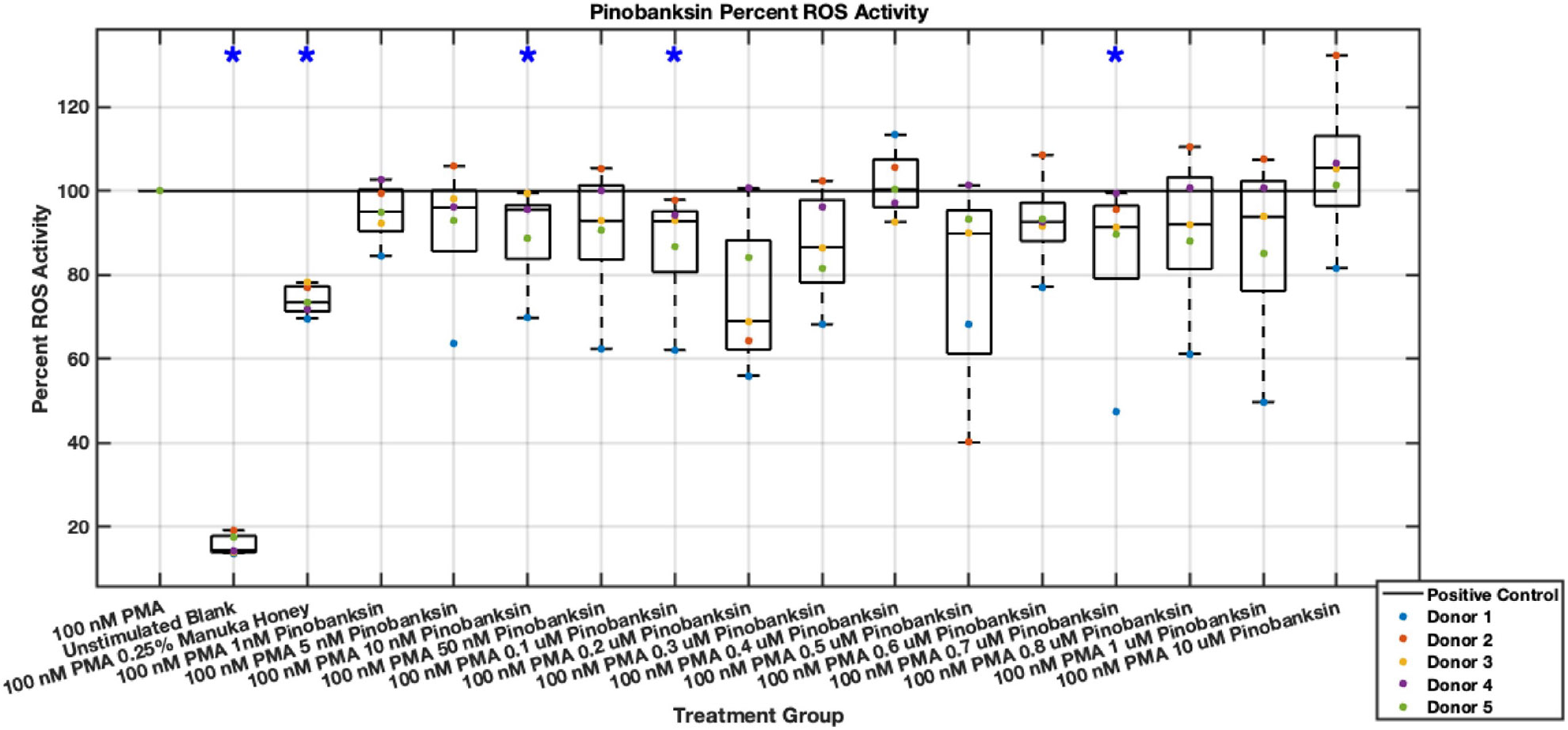
Effect of Pinobanksin on neutrophil intracellular ROS activity six hours post-PMA-stimulus. Dot plot points are represented as the mean of four wells for each five experiments (n = 5). Asterisk represents statistical significance from the 100 nM PMA positive control group. *p < 0.05.

**Fig. 19.**
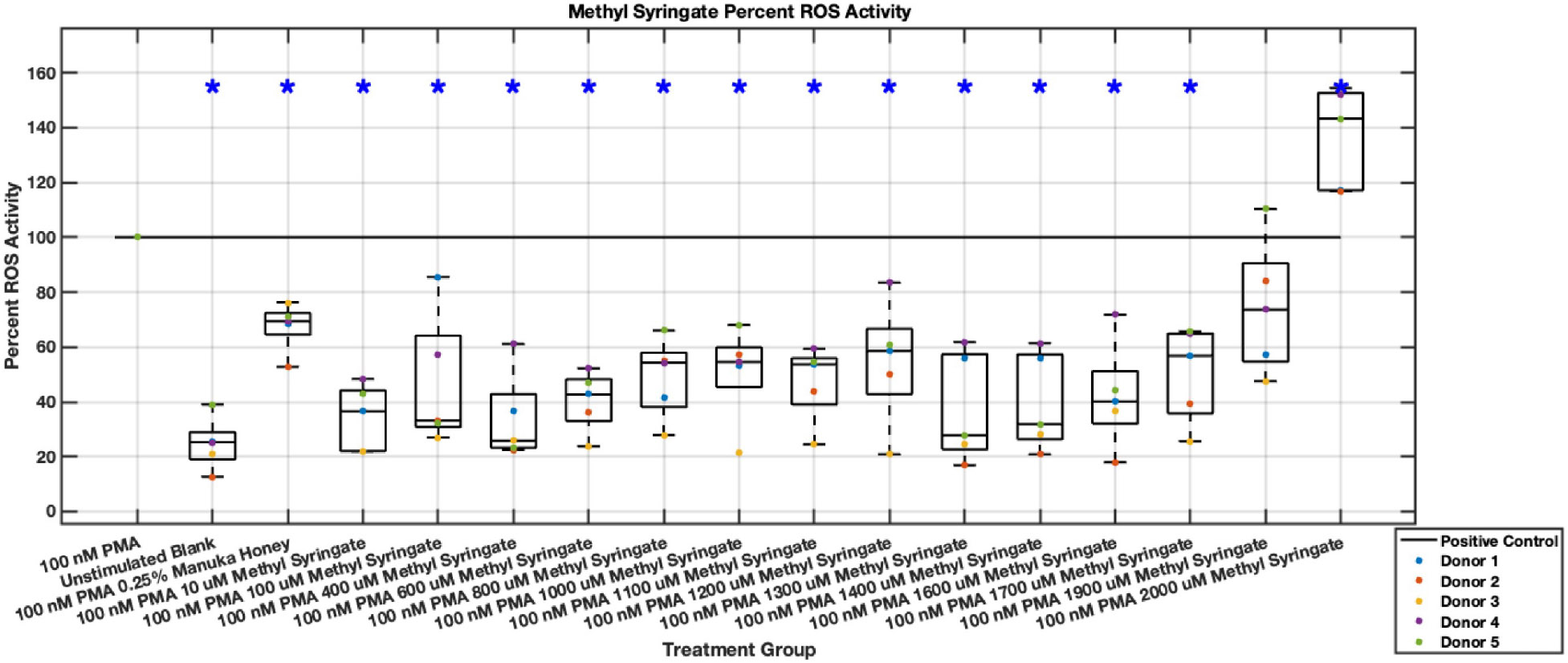
Effect of Methyl Syringate on neutrophil intracellular ROS activity three hours post-PMA-stimulus. Dot plot points are represented as the mean of four wells for each five experiments (n = 5). Asterisk represents statistical significance from the 100 nM PMA positive control group. *p < 0.05.

**Fig. 20.**
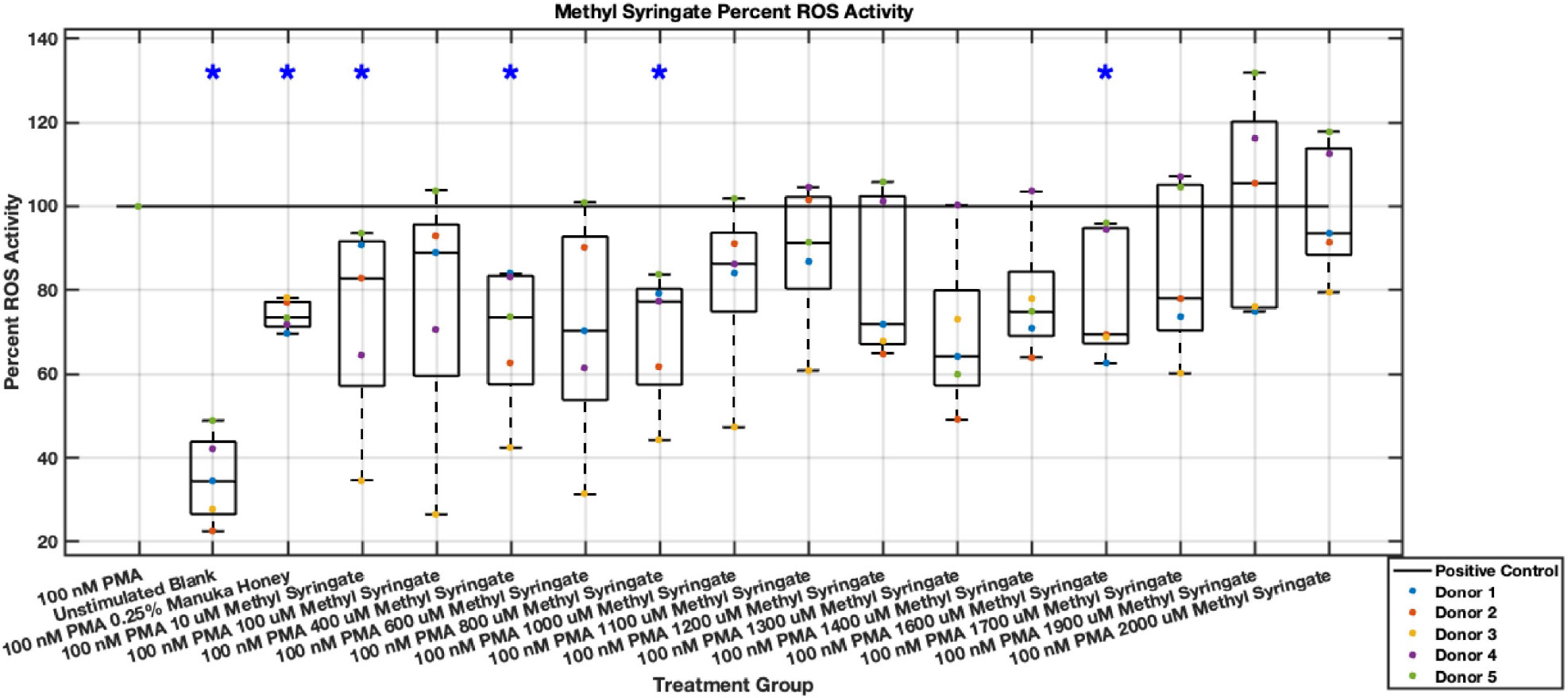
Effect of Methyl Syringate on neutrophil intracellular ROS activity six hours post-PMA-stimulus. Dot plot points are represented as the mean of four wells for each five experiments (n = 5). Asterisk represents statistical significance from the 100 nM PMA positive control group. *p < 0.05.

Methyl syringate inhibits intracellular ROS activity in stimulated neutrophils, while flavonoids chrysin, pinocembrin, and pinobanksin have diminished effect sizes (Figs. 13,14,15,16,17,18,19,20). Similarly to NET data, methyl syringate inhibits ROS activity in neutrophils by over 60% in some concentrations (Figs. 19,20). These data also indicate that the effect size compared to positive control decreases 6 hours after PMA stimulation. Chrysin, pinocembrin, and pinobanksin showed some promise with various concentrations showing statistically significant reductions, though with small effect size (median values were within 20% of positive control). For methyl syringate, however, the reduction in intracellular ROS activity demonstrated a similar therapeutic range as the NET data of 10–1700 μM with values ranging between 36% to 50% of the positive control ROS activity levels. These data correspond to a 50%–66% reduction in intracellular ROS activity due to methyl syringate treatment. At the highest concentration of methy syringate, 2 mM, a statistically significant increase in intracellular ROS activity was observed (Figs. 19,20) suggesting a therapeutic limit of methyl syringate.

Diminished statistical significance was found after the 6-hour time point. Additionally, higher concentrations of flavonoids had median ROS activity values statistically significantly higher than that of the positive control, suggesting a concentration limit in which the flavonoids may stimulate ROS activity within neutrophils. This effect was observed most strongly in pinocembrin, which showed statistical significance (Figs. 15,16).

### 3.2 Combination

#### 3.2.1 NET Formation

**Fig. 21.**
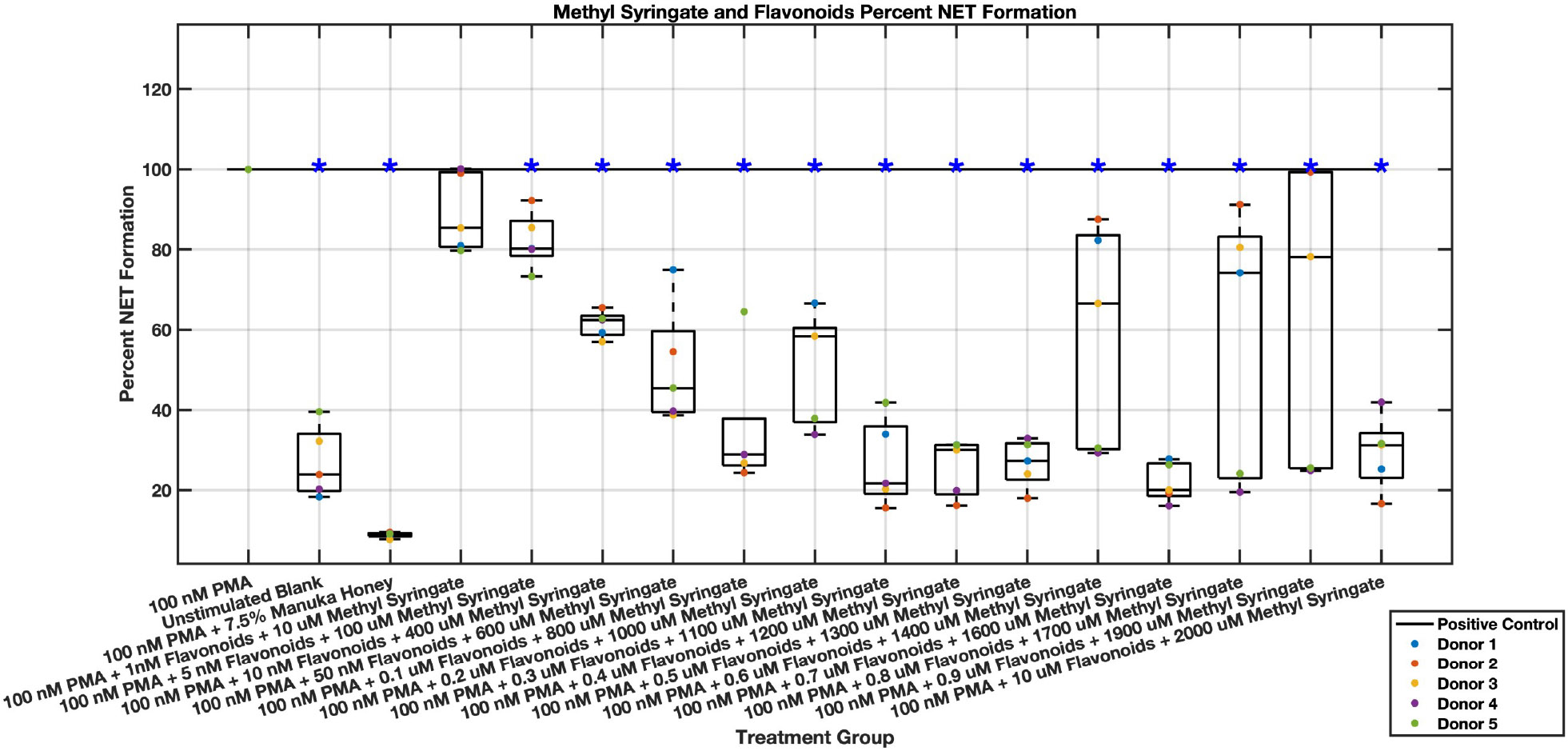
Effect of Methyl Syringate in combination with flavonoids chrysin, pinocembrin, and pinobanksin on neutrophil NET formation three hours post-PMA-stimulus. Dot plot points are represented as the mean of four wells for each five experiments (n = 5). Asterisk represents statistical significance from the 100 nM PMA positive control group. *p < 0.05.

**Fig. 22.**
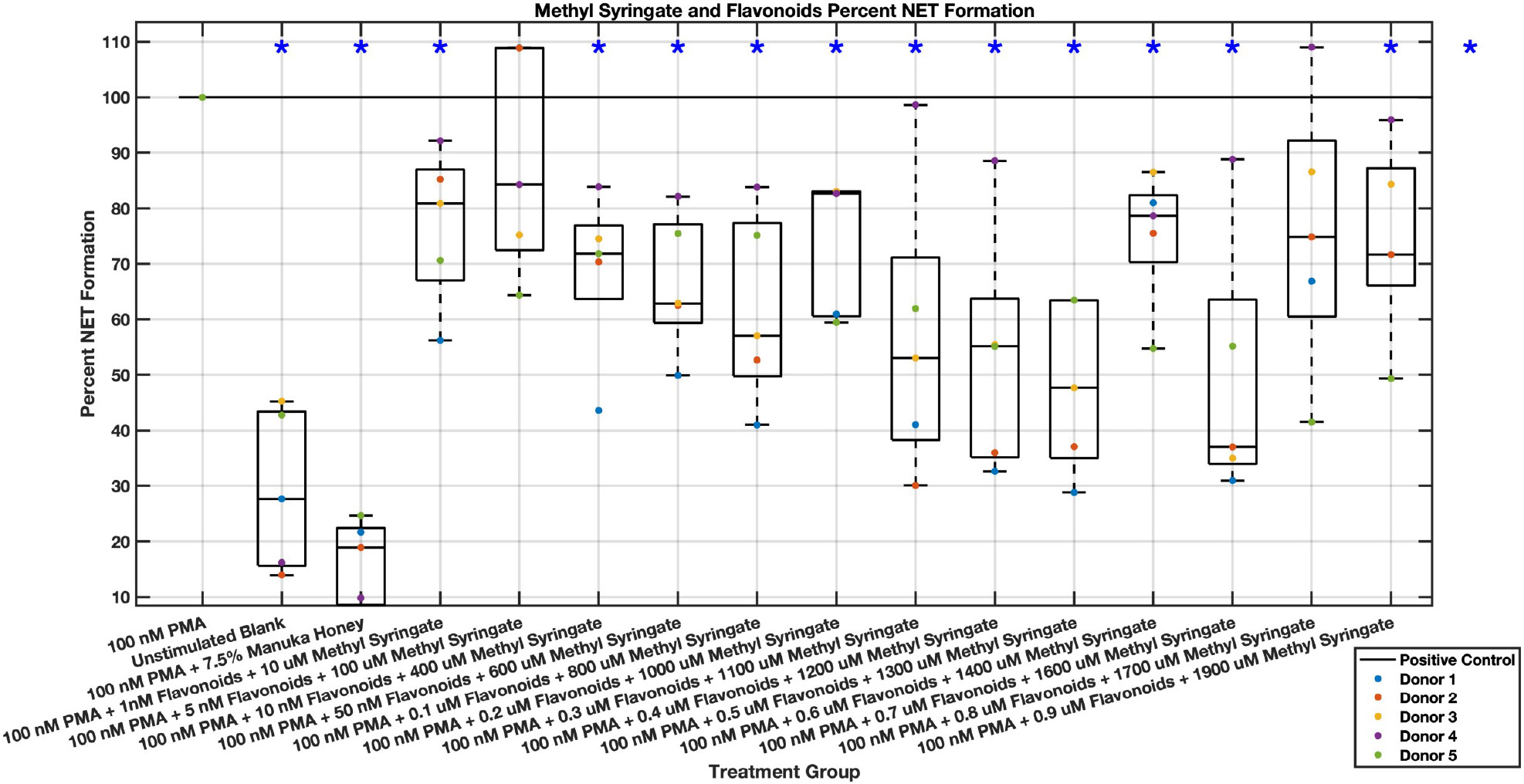
Effect of Methyl Syringate in combination with flavonoids chrysin, pinocembrin, and pinobanksin on neutrophil NET formation six hours post-PMA-stimulus. Dot plot points are represented as the mean of four wells for each five experiments (n = 5). Asterisk represents statistical significance from the 100 nM PMA positive control group. *p < 0.05.

A combination of flavonoid and phenolic compound dilutions inhibit NET formation with a large effect size but with high variability from subject to subject. Chrysin, pinocembrin, pinobanksin, and methyl syringate dilutions were combined and assayed to assess whether these compounds showed a synergistic or additive effect. A more substantial effect size was observed in NET formation compared to the positive control (Figs. 21,22); however, the data was less consistent, and a therapeutic range was more difficult to establish. Specific concentrations of the combination “cocktail” of flavonoids and methyl syringate reduced neutrophil NET formation to at or below baseline unstimulated control levels, suggesting a nearly complete inhibition of neutrophil NET-associated DNA release. These concentrations that showed near-total inhibition of PMA-induced NET formation were 0.3 μM chrysin, pinocembrin, pinobanksin, and 1100 μM methyl syringate, 0.4 μM chrysin, pinocembrin, pinobanksin, and 1200 μM methyl syringate, and 0.5 μM chrysin, pinocembrin, pinobanksin, and 1300 μM methyl syringate. A loss of statistical significance occurred at 0.6 μM chrysin, pinocembrin, pinobanksin, and 1400 μM methyl syringate, but 0.7 μM chrysin, pinocembrin, pinobanksin, and 1600 μM methyl syringate showed similar values around 20% NET levels compared to positive control. Additionally, 1 μM chrysin, pinocembrin, pinobanksin, and 2 mM methyl syringate had levels corresponding to 30% that of the positive control. Overall, this range of 10 nM chrysin, pinocembrin, pinobanksin, and 400 μM methyl syringate to 1 μM chrysin, pinocembrin, pinobanksin, and 2 mM methyl syringate had reductions in NET formation ranging from 40%–80%.

#### 3.2.2 ROS Activity

**Fig. 23.**
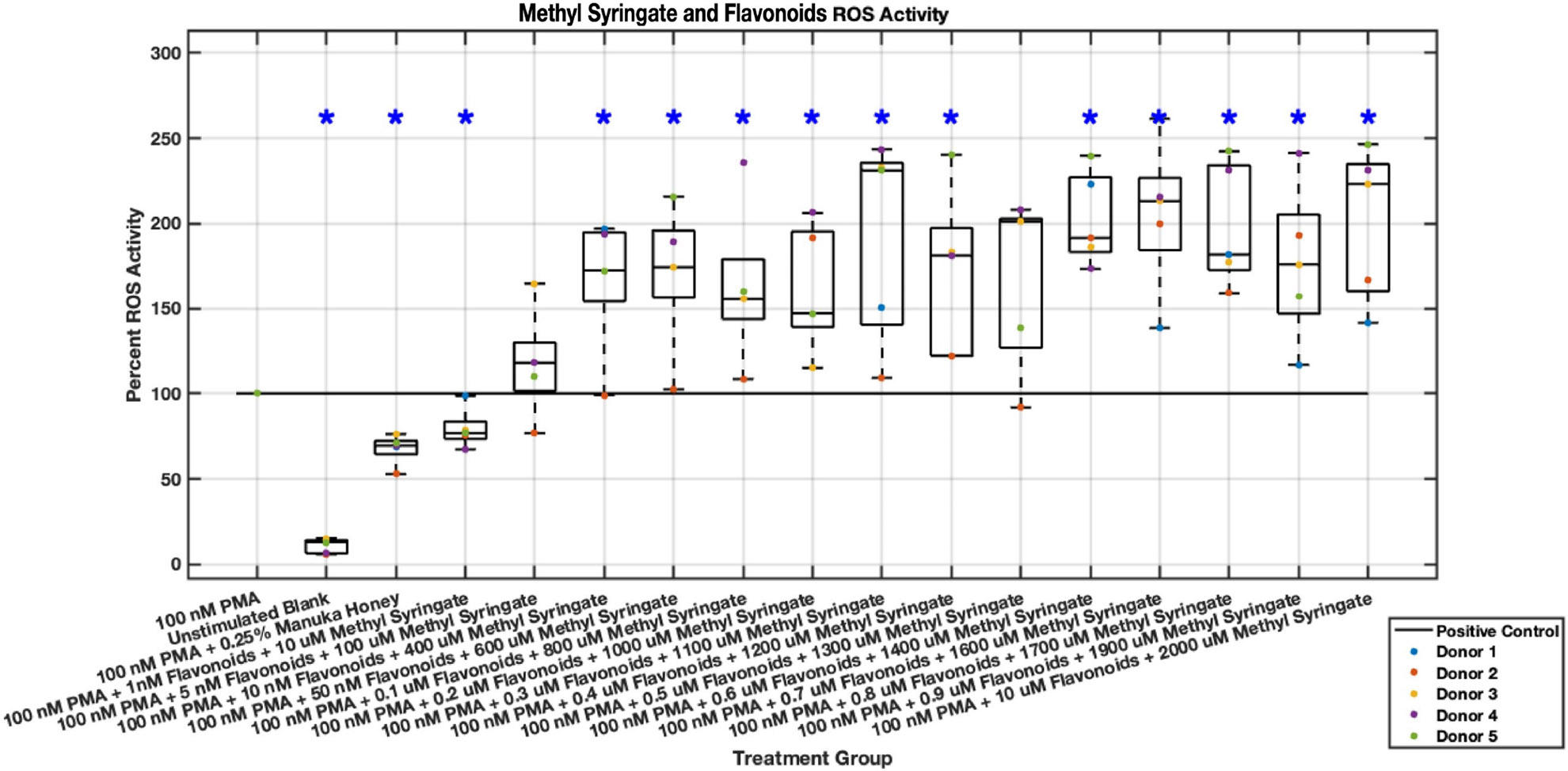
Effect of Methyl Syringate in combination with flavonoids chrysin, pinocembrin, and pinobanksin on neutrophil intracellular ROS activity three hours post-PMA-stimulus. Dot plot points are represented as the mean of four wells for each five experiments (n = 5). Asterisk represents statistical significance from the 100 nM PMA positive control group. *p < 0.05.

**Fig. 24.**
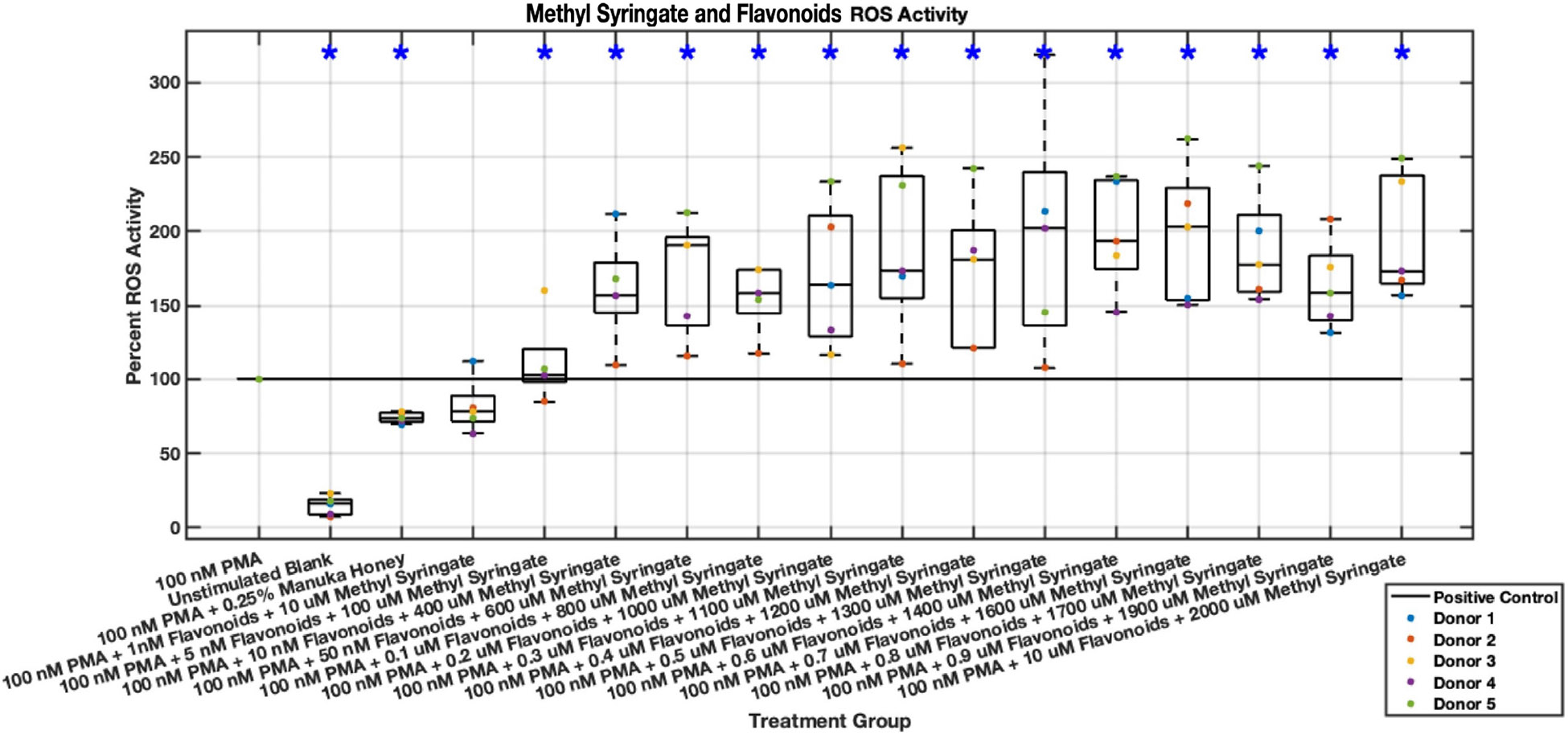
Effect of Methyl Syringate in combination with flavonoids chrysin, pinocembrin, and pinobanksin on neutrophil intracellular ROS activity six hours post-PMA-stimulus. Dot plot points are represented as the mean of four wells for each five experiments (n = 5). Asterisk represents statistical significance from the 100 nM PMA positive control group. *p < 0.05.

While promising results were demonstrated by a combination of methyl syringate and the three flavonoids regarding NET formation, inverse results were shown regarding intracellular ROS activity (Figs. 23,24). The inclusion of all three flavonoids and methyl syringate increased ROS activity in stimulated neutrophils by nearly twice in some concentrations, indicating that this combination “cocktail” may be an inducer of ROS activity. Data values from 5 nM chrysin, pinocembrin, pinobanksin, and 100 μM methyl syringate to 1 μM chrysin, pinocembrin, pinobanksin, and 2 mM methyl syringate indicated ranges of increased ROS activity between 120% and nearly 200% compared to PMA-stimulated positive control.

### 3.3 Whole Manuka Honey

#### 3.3.1 NET Formation

**Fig. 25.**
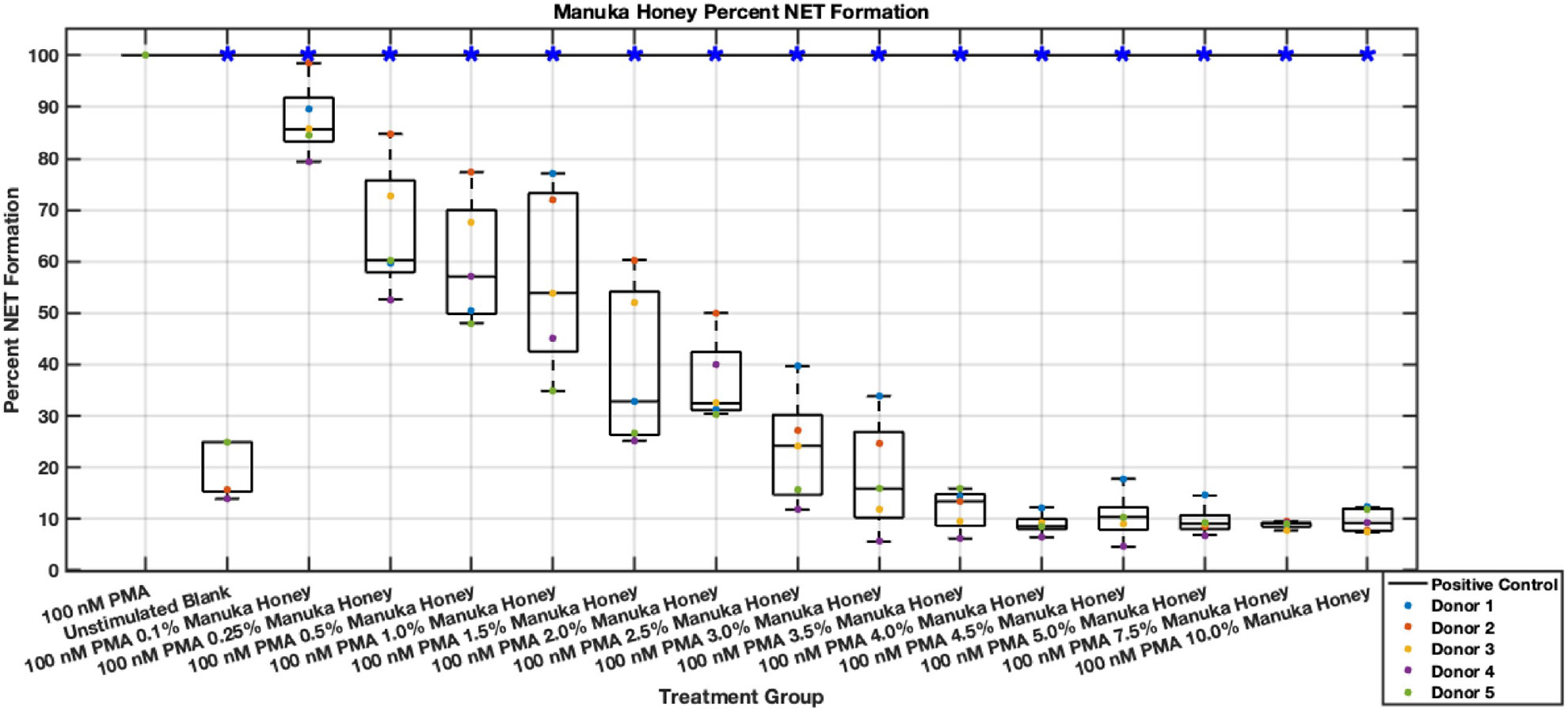
Effect of whole Manuka honey on neutrophil NET formation three hours post-PMA-stimulus. Dot plot points are represented as the mean of four wells for each five experiments (n = 5). Asterisk represents statistical significance from the 100 nM PMA positive control group. *p < 0.05.

**Fig. 26.**
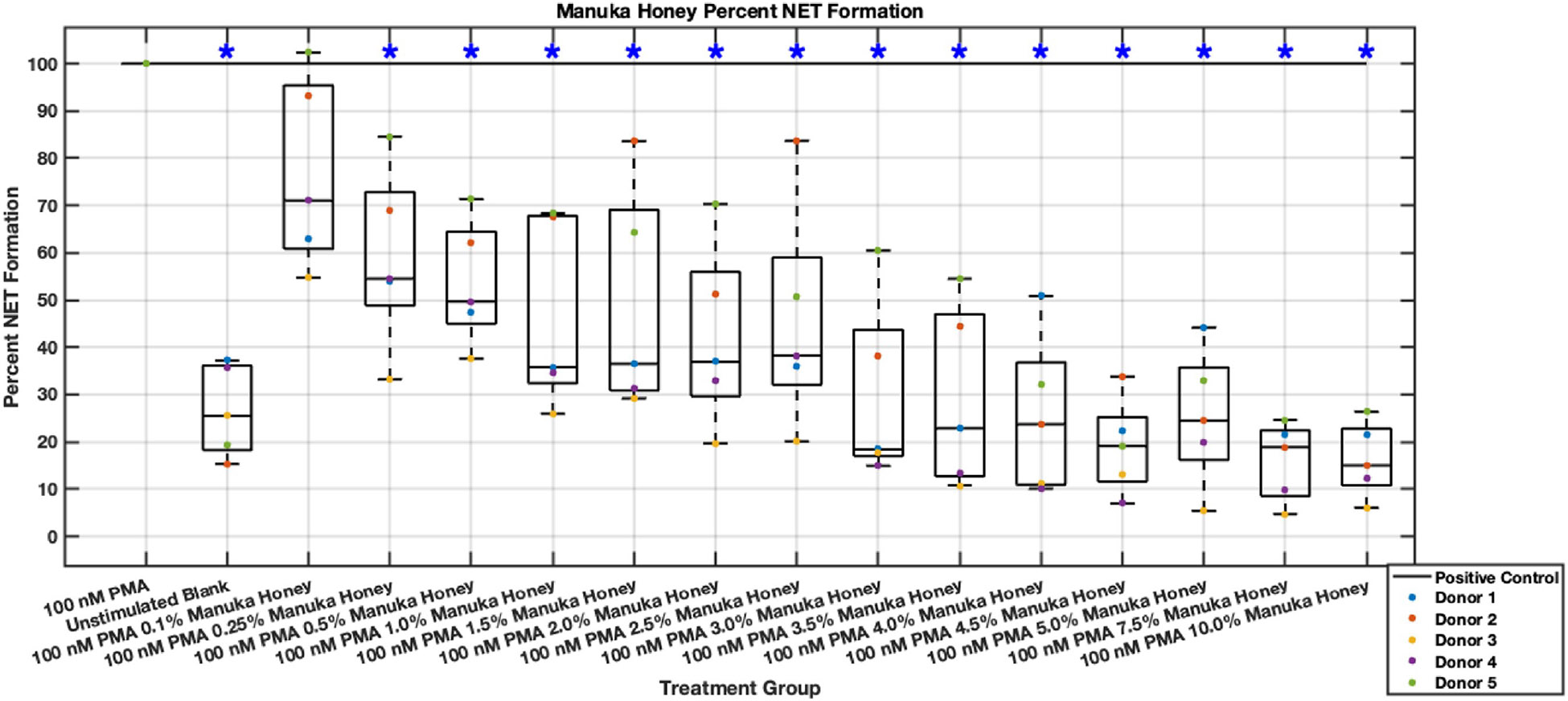
Effect of whole Manuka honey on neutrophil NET formation six hours post-PMA-stimulus. Dot plot points are represented as the mean of four wells for each five experiments (n = 5). Asterisk represents statistical significance from the 100 nM PMA positive control group. *p < 0.05.

Manuka honey shows a potent dose-dependent inhibition of NET-associated DNA release, which remains consistent even after 6 hours post-stimulus. In contrast with other data sets from this investigation, whole Manuka honey is the only bioactive that achieved long-lasting inhibition of neutrophil inflammatory behavior. Reductions near or below baseline activation due to cell culture conditions (negative controls) were observed in the 3–10% range in both 3- and 6-hour plates (Figs. 25,26). Even low concentrations starting at 0.5% Manuka honey showed statistically significant NET release reductions after 3 hours post-stimulus. Interestingly, at the 10% concentration, no sign of cytotoxicity was observed in terms of NET release, which is in disagreement with prior reports [12]. The range of 3.5% and 10% Manuka honey boasted the highest reductions in NET formation levels, with data values at 10% of positive control. Additionally, the 6-hour data showed statistically significant reductions with levels at or below 30% of positive control levels. Thus, Manuka honey demonstrated lasting inhibition of NETosis between 3–10% Manuka honey.

#### 3.3.2 ROS Activity

**Fig. 27.**
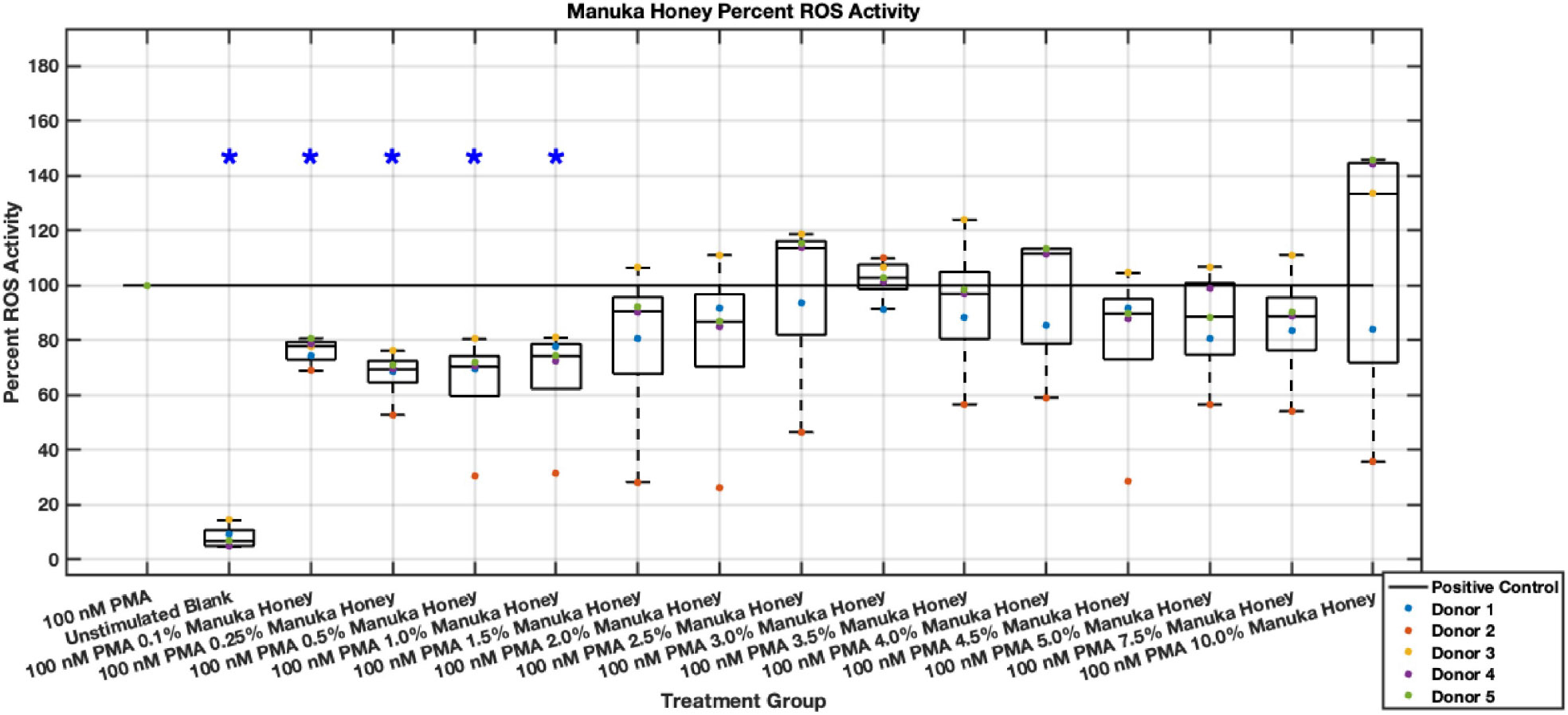
Effect of whole Manuka honey on neutrophil intracellular ROS activity three hours post-PMA-stimulus. Dot plot points are represented as the mean of four wells for each five experiments (n = 5). Asterisk represents statistical significance from the 100 nM PMA positive control group. *p < 0.05.

**Fig. 28.**
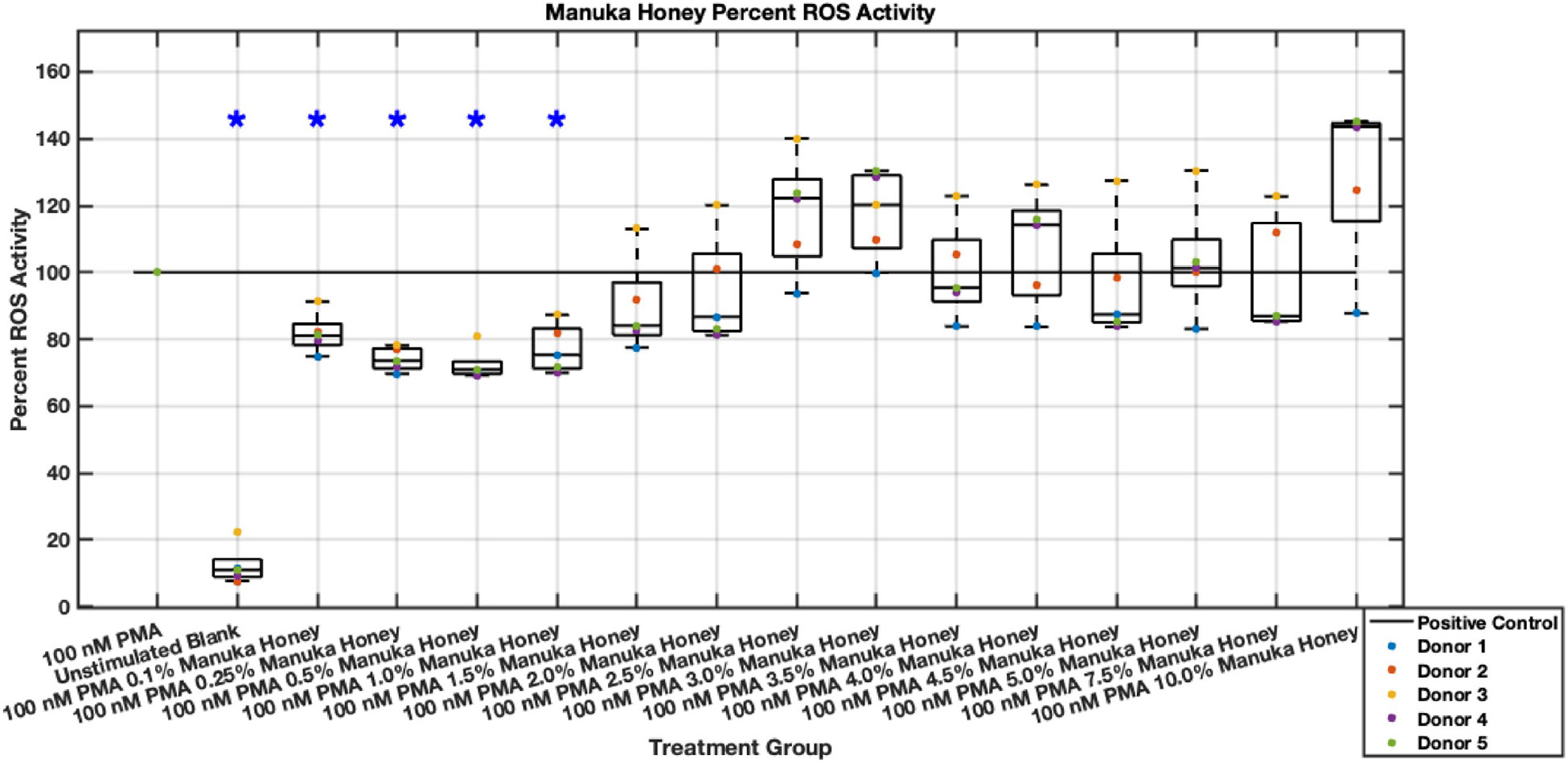
Effect of whole Manuka honey on neutrophil intracellular ROS activity six hours post-PMA-stimulus. Dot plot points are represented as the mean of four wells for each five experiments (n = 5). Asterisk represents statistical significance from the 100 nM PMA positive control group. *p < 0.05.

In contrast with the promising results regarding Manuka honey’s inhibition of neutrophil NET release, Manuka honey only demonstrated a statistically significant reduction in ROS at a 0.1% to 1% concentration range with a low effect size range of approximately 25% to 35% (Figs. 27,28). However, these ROS inhibitions at lower concentrations were maintained through the six-hour timepoint. Manuka honey concentrations higher than 2.5% began trending towards values similar to positive controls, with 10% Manuka honey stimulating ROS after six hours. These data suggest that the higher concentrations of Manuka honey may stimulate ROS activity instead of inhibiting it, which lends itself to previously established cytotoxic limits [14].

## 4. Discussion

The present study sought to elucidate which compounds in Manuka honey show the most promise in reducing neutrophil inflammatory behavior. This study was confined to the focus of ROS activity and NET formation in response to PMA stimulus. The data generated by these investigations concluded that methyl syringate provides a potent reduction in neutrophil ROS activity and NET release within the wide range of 100–1700 μM. Significantly, it was determined that Manuka honey-derived flavonoids chrysin, pinocembrin, and pinobanksin had little to no effect on ROS activity or NET formation when in isolated form. When combined in a “cocktail”, these components increased ROS activity. However, the combination of methyl syringate and flavonoids lowered NET levels, likely due to the inclusion of methyl syringate as reductions showed marked similarity in pattern and magnitude to the data from isolated methyl syringate. Whole Manuka honey showed tremendous effectiveness in reducing NETosis but modest effectiveness in smaller doses at reducing ROS activity and stimulated ROS activity at higher doses. This study thus concluded that chrysin, pinocembrin, and pinobanksin are not largely responsible for reductions in ROS activity and NET release in pro-inflammatory stimulated neutrophils. The study, however, did elucidate the primary compound responsible for Manuka honey’s anti-NETosis capabilities, methyl syringate. Methyl syringate also showed the capacity to reduce ROS activity even further than whole Manuka honey, indicating that there may be components of Manuka honey that counteract or impede methyl syringate’s antioxidant abilities. Medical grade Manuka honey is often characterized by its methylglyoxyl (MGO) concentration, which correlates to its potency as an antimicrobial therapeutic [51]. The findings of this study demonstrate that perhaps the concentration of methyl syringate should thus be used to characterize Manuka honey’s anti-inflammatory therapeutic properties.

Great promise has been shown by Manuka honey in reducing inflammation and preventing infection, but the resounding questions as to why and how Manuka honey works have previously not been addressed. Methylglyoxal has been characterized as the primary chemical component responsible for Manuka honey’s antimicrobial properties. However, the other half of what makes Manuka honey so promising, its capabilities to reduce inflammation, remains unclear [52]. This expansive and detailed study was designed to split Manuka honey into its constituent flavonoid and phenolic fractions and investigate their effects on two key neutrophil inflammatory behaviors that impede host-biomaterial integration [2]. While methyl syringate has been measured and somewhat characterized in Manuka honey, this is presently the first study to indicate its role in Manuka honey’s anti-inflammatory potential.

This study also looked at whole Manuka honey versus its isolated compounds in combination to determine potential synergistic or additive effects and compare them to their effects within whole Manuka honey. It was then determined that these components act best together when they are within whole Manuka honey and that a combination “cocktail” of the most prevalent candidates for Manuka honey’s anti-inflammatory action is ineffective. Overall, these are the two most impactful findings of this study. First, methyl syringate is responsible for reducing two significant neutrophil inflammatory behaviors and likely is the primary antioxidant and anti-inflammatory component of Manuka honey. Second, whole Manuka honey inhibits NET formation and ROS activity more than the isolates that constitute it. Thus, “the whole is greater than the sum of its parts”.

Painstaking care was taken to remove extraneous variables such as donor-to-donor variability, with separate experiments and controls being conducted and recording spanning five different blood donors, with each experiment being normalized to it’s own controls. Additionally, strict sterility was enforced to prevent the activation of neutrophils outside the intended PMA stimulus. Despite these factors being considered, neutrophil data is often widely variable [53]. In this study, different responses and data trends existed from donor to donor, with some responding more strongly to different components and concentrations than others. Thus, when all data were combined into one data set, some regions of the data had large standard deviations. This effect can be seen in the higher ranges of Manuka honey ROS activity, wherein the stimulatory effect on ROS activity was more robust in some donor neutrophils than others.

Donor to donor variability is to be expected, especially with primary neutrophils. However, different donor data sets showed different trends, with effective dose ranges and effect sizes occurring in different patterns and at different concentration ranges. This effect was most prominently seen within the combination of the three flavonoid and methyl syringate combination experiments at higher concentrations. These individual donor data sets, when combined, yielded non-progressive and seemingly random trends. The pharmacokinetics of the data visualized in Fig. 21 (NETosis at the three hour time point in response to all three flavonoids and methyl syringate) indicate that as the concentration of all three flavonoids and methyl syringate increase from 1 nM of flavonoids and 10 μM methyl syringate to 0.1 μM of flavonoids and 800 μM methyl syringate, there is a steady, linear decrease in NETosis which plateaus in the subsequent concentrations. This plateau holds steady in a consistent manner across all donors until 0.6 μM flavonoids and 1400 μM methyl syringate, where donor 4 and 5 remain in this plateau, the other three donors start to demonstrate increasing diminishing effects on NETosis compared to the positive control. This diminished effect size for the separate donors is observed in the 0.8 μM flavonoids and 1700 μM methyl syringate data set and the 0.9 μM flavonoids and 1900 μM methyl syringate data set. Overall, these three data sets are still significantly different from the positive control, and not significantly different from the surrounding data sets, forming that the plateau of maximal effect still remains consistent through this concentration range. Additionally, experimental errors must be considered. Due to the large amount of sample groups, experiments, and repetitions, the potential for pipetting errors cannot be ruled out. Fluid shear stress is an activating factor for neutrophils and thus pipetting must be done very delicately [54]. Variation in the data could likely occur due to shear stress being applied to the neutrophils during seeding of the plates. Additionally, the solubility of the flavonoids could be a limiting factor, where as concentrations increase, especially when combined, there is still some small amount of insolubilized precipitate which could activate neutrophils or decrease the effectiveness of the flavonoids.

Neutrophils, even within their physiological environment, are classically considered to have a half-life of approximately 13–19 hours [55]. This lifespan becomes even shorter when outside the body. However, these half-life values are controversial [56,57]. These life spans are becoming more widely ranging and nuanced as new studies are being performed in different contexts [58]. Thus temporal dynamics of *in-vitro* neutrophil models become difficult to perform. As seen in the 6-hour data from this study, variability in neutrophil behavior becomes problematic after six hours post-stimulus. Thus, this study was limited to three and six-hour time points. The temporal dynamics of these compounds are of interest and are certainly worth further investigation, but they were outside the scope of this study.

Further, this study was limited in scope to the two neutrophil responses of ROS activity and NET formation due to the ambitious goal of detail and breadth of the compounds studied, the concentrations of those compounds, and the high number of experiments conducted per investigation to have appropriate sample sizes. Neutrophil proinflammatory signaling and the release of degradative enzymes is a crucial component of their behavior in response to implanted biomaterials. Future studies will delve into the effects of methyl syringate and Manuka honey on these factors.

Surprisingly, there was no increase in NETosis from the 10% concentration of Manuka honey, which disagrees with prior reports [12]. However, intracellular increases of ROS activity increased, approaching the higher concentrations of Manuka honey. Thus, a cytotoxic limit is still implicated through a different modality than previously established. The lowered ability of chrysin, pinobanksin, and pinocembrin to reduce intracellular ROS activity and NETosis was surprising, as previous pilot study data from a differentiated HL60 cell line suggested strong potential from these components. Antioxidant mechanisms of action can vary. Some antioxidants directly decrease oxidative damage by interacting with the ROS and neutralizing it or by reducing the expression or activity of intracellular ROS-generating enzymes [59]. It is possible that chrysin, pinocembrin, and pinobanksin may have more potent antioxidant effects, but these effects occur extracellularly. Thus, the potential of these components should not be discarded outright. Future studies should be performed regarding their extracellular mechanisms, as the scope of this investigation was on intracellular ROS activity.

It was also unexpected that combining flavonoids and methyl syringate concentrations would stimulate ROS activity, although this is in line with ROS activity stimulation of larger concentrations of Manuka honey. These statistically significant changes beginning at 10 nM flavonoids and 400 μM methyl syringate are potentially due to an overabundance of antioxidants within the microenvironment. The increase on ROS activity is in line with prior reports involving overdose intake of antioxidant compounds, which demonstrate raised levels of ROS activity as well as diminished levels of antioxidant enzyme superoxide dismutase (SOD) and detoxified enzyme glutathione S-transferases (GST) [60]. Interestingly, these results were not observed in whole Manuka honey, perhaps because other compounds within the honey slow the release of chrysin, pinocembrin, and pinobanksin, creating a more extended release profile. The increase in ROS activity from higher levels of individual flavonoids could also be explained by this reasoning. Perhaps then, overwhelming the cells with multiple compounds all at once may be deleterious, and having these compounds sequestered within whole Manuka honey is more effective. After the study, CyQUANT MTT Cell Viability Assay (Invitrogen Ref. No. V13154) was performed according to manufacturer instructions on the combination of flavonoid and methyl syringate concentrations using a differentiated HL60 cell line as a monoclonal analog for reproducibility due to lower patient-to-patient variability [13,23,61-63]. The data from this preliminary study suggest that there is in fact a mild reduction in viability (~20%) at the highest concentration of all flavonoids and methyl syringate. Full methods, data sets, graph, and statistical analysis are available by request of the author.

The goal of the present study was two-fold. The first objective was to determine which chemical component of Manuka honey was responsible for its demonstrated antiinflammatory properties. The findings of this study are that methyl syringate is responsible. The second objective was to determine whether these components work best outside of the high-glucose content of Manuka honey, in some combination “cocktail”, or if whole Manuka honey is optimal. Whole Manuka honey was more effective at reducing NETosis and was less stimulatory of intracellular ROS activity than chrysin, pinocembrin, pinobanksin, and methyl syringate in combination.

Manuka honey is highly promising as a biomaterial additive because it prevents infection in the implant microenvironment and reduces inflammation. Utilizing the demonstrated ability of Manuka honey to both prevent infection and ameliorate the acute phase of inflammation, we aim to develop the potential of Manuka honey as an additive for biomaterial applications to prevent fibrous encapsulation and device failure. This study’s findings further emphasize Manuka honey’s ability to lower neutrophilmediated inflammation and highlight the primary compound responsible.

It can be concluded from this study that methyl syringate warrants further investigation into antioxidant and anti-inflammatory properties. It can also be further concluded that Manuka honey serves as a better candidate for a therapeutic than its isolated components. Future investigation will be conducted into whether Manuka honey with added methyl syringate will be more effective at lowering intracellular ROS levels than whole Manuka honey alone. The third main category of neutrophil-mediated inflammation is their potent signaling capabilities to recruit immune cells to the implant environment and prime them to proinflammatory phenotypes. Further studies are underway to assess Manuka honey and methyl syringate’s ability to quell these signals and promote pro-remodeling and revascularization signals from neutrophils.

Future work is required to further elucidate methyl syringate’s ability to lower NETosis and intracellular ROS activity. To understand what is occurring at an intracellular level to lower NETosis and intracellular ROS activity, indepth cell signaling studies should be performed. Mechanistic studies should also be performed investigating the interactions between these compounds to determine if this has an effect on their efficacy. Additionally, the effects of methyl syringate should be assayed in a more complex, simulated host-biomaterial microenvironment, involving a coculture of multiple cell lines. These findings should then be validated in *in vivo* experiments and then move forward into clinical studies. Furthermore, methyl syringate, or Manuka honey with added methyl syringate content, should be incorporated into biomaterials. The release profile of methyl syringate as well as Manuka honey should be assayed, and the efficacy of Manuka honey or methyl syringate-laden biomaterials in quelling the neutrophil inflammatory response to implanted biomaterials should be studied.

The results of these future studies could help elucidate the ability of these bioactives to improve clinical outcomes in patients receiving biomaterial implants. For example, clinical outcomes of patients receiving small diameter vascular grafts, especially in below the knee or below the elbow applications are poor due to fibrous encapsulation, which has been strongly linked to the neutrophil-predominant acute phase of inflammation [2,4,5]. The endstage fibrotic capsule has been demonstrated as being nonuniformly constrictive, causing flow disturbances within the vessel, causing increased thrombosis. The capsule is also nonelastic, causing a discrepancy between the mechanical properties of the vasculature and the device potentially causing hyperplasic cell proliferation [64]. Additionally, the avascularity of the device due to the fibrous capsule will prevent full healing and reendothelialization of the vessel [65]. Finally, the sustained oxidative and enzymatic degradation by neutrophils and macrophages due to chronic unresolved inflammation can directly lead to device failure and rupture. Small diameter grafts are far more prone to failure from these mechanisms because even small occlusions, flow rate disturbances, or mechanical mismatches can greatly affect the total flow through such a small diameter.

Thus, by targeting the initial immune response from neutrophils at the site of implantation, clinical outcome of biomaterial implants could be improved. Lowering the NET release and ROS activity at the host-biomaterial interface would lower the chances of developing chronic inflammation and sustained immune attack of the biomaterial implant site. These effects would thus prevent neutrophilmediated fibrosis and device failure. This study is among the first steps in establishing that methyl syringate indeed drives two of Manuka honeys primary anti-inflammatory capabilities. This study also establishes a therapeutic window for methyl syringate, to be further refined and specified in future works.

## 5. Conclusions

Manuka honey has shown great promise in wound healing both by its antibacterial properties, but also by its anti-inflammatory capabilities. However, the specific mechanisms, components, and most effective compositions are not fully understood and require further research. The investigation presented in this study sought to elucidate further what makes Manuka honey so promising as an antiinflammatory therapeutic. Overall, this study highlights the importance of methyl syringate in Manuka honey’s demonstrated ability to quell neutrophil inflammatory behavior. Further studies are in progress to assess methyl syringate and whole Manuka honey’s effect on neutrophil inflammatory signaling, degradative enzyme release, and pro-remodeling factors. Additionally, further studies are needed to determine if Manuka honey with increased levels of methyl syringate could be a viable improvement in the therapeutic qualities of Manuka honey.
